# Toward Water-Resistant, Tunable Perovskite Absorbers Using Peptide Hydrogel Additives

**DOI:** 10.1021/acsaem.4c01089

**Published:** 2024-09-13

**Authors:** Tom Flavell, Dawei Zhao, Fahad A. Aljuaid, Xuzhao Liu, Alberto Saiani, Alexei B. Preobrajenski, Alexander V. Generalov, Ben F. Spencer, Alex S. Walton, Andrew G. Thomas, Wendy R. Flavell

**Affiliations:** † Photon Science Institute, University of Manchester, Oxford Road, Manchester, M13 9PL, United Kingdom; ‡ Department of Physics and Astronomy, University of Manchester, Oxford Road, Manchester, M13 9PL, United Kingdom; § Department of Materials, University of Manchester, Oxford Road, Manchester, M13 9PL, United Kingdom; ∥ Manchester Institute of Biotechnology, University of Manchester, Oxford Road, Manchester, M13 9PL, United Kingdom; ⊥ Division of Pharmacy and Optometry, School of Health Sciences, University of Manchester, Oxford Road, Manchester, M13 9PL, United Kingdom; # MAX IV Laboratory, Lund 221 00, Sweden; ∇ Henry Royce Institute, University of Manchester, Oxford Road, Manchester, M13 9PL, United Kingdom; ○ Department of Chemistry, University of Manchester, Oxford Road, Manchester, M13 9PL, United Kingdom

**Keywords:** perovskites, methylammonium lead iodide perovskites, peptide additives, near-ambient pressure X-ray photoelectron spectroscopy, nanoparticles

## Abstract

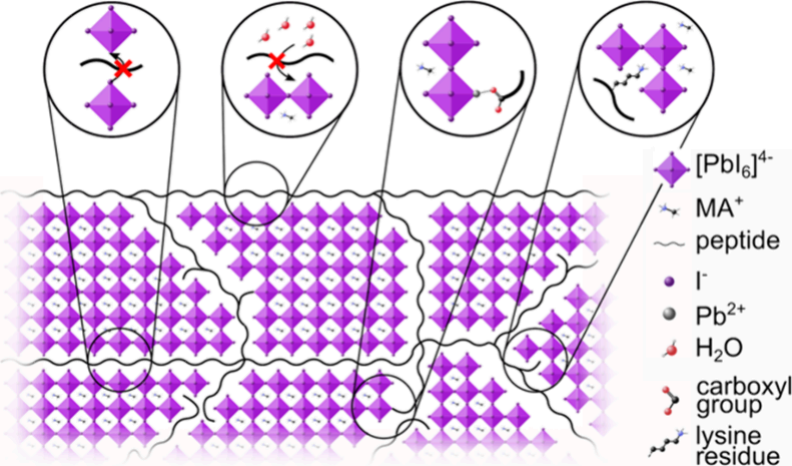

In recent years, hydrogels have been demonstrated as simple and cheap additives to improve the optical properties and material stability of organometal halide perovskites (OHPs), with most research centered on the use of hydrophilic, petrochemical-derived polymers. Here, we investigate the role of a peptide hydrogel in passivating defect sites and improving the stability of methylammonium lead iodide (MAPI, CH_3_NH_3_PbI_3_) using closely controlled, *in situ* X-ray photoelectron spectroscopy (XPS) techniques under realistic pressures. Optical measurements reveal that a reduction in the density of defect sites is achieved by incorporating peptide into the precursor solution during the conventional one-step MAPI fabrication approach. Increasing the concentration of peptide is shown to reduce the MAPI crystallite size, attributed to a reduction in hydrogel pore size, and a concomitant increase in the optical bandgap is shown to be consistent with that expected due to quantum size effects. Encapsulation of MAPI crystallites is further evidenced by XPS quantification, which demonstrates that the surface stoichiometry differs little from the expected nominal values for a homogeneously mixed system. *In situ* XPS demonstrates that thermally induced degradation in a vacuum is reduced by the inclusion of peptide, and near-ambient pressure XPS (NAP-XPS) reveals that this enhancement is partially retained at 9 mbar water vapor pressure, with a reduced loss of methylammonium (MA^+^) from the surface following heating achieved using 3 wt % peptide loading. A maximum power conversion efficiency (PCE) of 16.6% was achieved with a peptide loading of 3 wt %, compared with 15.9% from a 0 wt % device, the former maintaining 81% of its best efficiency over 480 h storage at 35% relative humidity (RH), compared with 48% maintained by a 0 wt % device.

## 1 Introduction

Since they were first demonstrated as viable photovoltaic (PV) materials by Miyasaka and co-workers in 2009, organometal halide perovskites (OHPs) have harbored enormous interest from the research community and are considered one of the most promising materials for use in next-generation solar cells.^1^ Unique optical and electronic properties such as tunable band gaps, strong light-absorption coefficients, high defect tolerance and low nonradiative recombination rates make OHPs compelling candidates for use as both absorbers and charge transporters in thin-film devices.^2−4^ Impressive advances in OHP research over the past decade have seen power conversion efficiencies (PCEs) of perovskite solar cells (PSCs) rise rapidly to 26.1%,^5^ rivalling that of current silicon-based devices, with a significant reduction in cost due to solution-based, low temperature fabrication processes.^1,2,4^

However, there remains a significant hurdle to overcome before PSCs are considered a feasible alternative to current PV technologies. OHPs exhibit poor stability to external factors such as moisture, heat, oxygen and illumination, which result in compositional changes that negatively affect the performance of PSCs.^6−8^ Methylammonium lead iodide (MAPI, CH_3_NH_3_PbI_3_), the archetypal OHP, undergoes irreversible degradation to nonphotoactive lead iodide (PbI_2_) on exposure to moisture and heat. Under these conditions, MAPI experiences a total loss of nitrogen from the surface in the form of ammonia gas (NH_3_) and partial loss of iodine in the form of hydrogen iodide (HI) and methyl-iodide (CH_3_I) in the cases of moisture-induced and thermal degradation respectively, as outlined in eqs 1 and 2.^6−8^
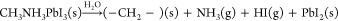
1
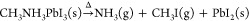
2Various routes have emerged to boost PSC longevity. Material-focused strategies, including ion substitution, surface passivation and additive engineering, and broader changes to device architecture, such as interface engineering, contact material optimization and device encapsulation, have brought PSCs closer to the operational lifetimes required for commercial deployment.^9−13^

Peptides with both amino (−NH_2_) and carboxylic (−COOH) groups have previously been used as capping ligands to effectively passivate MAPbBr_3_ (CH_3_NH_3_PbBr_3_) perovskite nanocrystals, with the amino and carboxylic groups passivating undercoordinated surface Br^–^ and Pb^2+^ respectively.^14^ Several recent studies have demonstrated the benefits of using amino acids and proteins to passivate perovskite nanocrystals.^15,16^ Lang et al. demonstrated that the introduction of lysine during the formation of MAPbBr_3_ crystals resulted in a hybrid unit cell, with the NH_3_^+^ groups in the amino acid substituting two methylammonium ions (MA^+^, CH_3_NH_3_^+^), evidenced by a lattice contraction. This new hybrid perovskite exhibited an increased band gap and an improved stability in water, the latter determined using an electrochemical approach.^17^

With respect to thin film PSCs, the introduction of amine- and carboxyl-functionalized materials, both as precursor additives and surface layers, has been shown to enhance and stabilize the PCE of devices. This improvement is largely associated with the suppression of charged defects via electrostatic iterations with the – NH_2_ and – COOH functional groups.^18−20^ It has been shown that C=O groups exhibit strong Lewis base-acid interactions with undercoordinated Pb^2+^, reducing antisite Pb_I_ defects (I site substitution by Pb), thereby reducing nonradiative recombination.^20,21^ On top of this, it has also been reported that aromatic structures can reduce neutral iodine defects.^22^

Some studies involving amino acids and peptides have centered on their use as modifiers for the electron transport layer (ETL)/perovskite interface. Due to the propensity for carboxylic groups to bond with the surface of titanium dioxide (TiO_2_) and the passivation of undercoordinated surface ions by the amino groups, commented on previously, amino acids have been shown to improve perovskite surface coverage on mesoporous ETL materials while also improving crystal growth.^11,23,24^ Devices utilizing this interfacial engineering have been shown to exhibit improved photovoltaic performance, attributed to enhanced charge transfer at the ETL/perovskite interface and reduction in trap states within the perovskite layer.^11,24^

Here, we explore whether the advantages of peptide passivation may be combined with 3D hydrogel formation to achieve both passivation (in wet and dry environments) and particle size control (leading to optical tunability). As reported in our previous work^12^ and that of others,^25,26^ the introduction of hydrogel additives to the perovskite precursor solution can be a simple and effective means by which to influence crystal growth, passivate defect sites and improve stability and device performance. Compared with petrochemical-derived synthetic polymer hydrogels, including poly(2-hydroxyethyl methacrylate) (pHEMA) studied in our previous work,^12^ peptide hydrogels synthesized from amino acids represent a more sustainable route to achieving highly stable OHPs.^27^ However, to date, to the best of our knowledge, little research into peptide hydrogel additives has been conducted and no in-depth study of the surface chemistry of such composites has been provided.

In this work, we study the influence of a peptide hydrogel additive on the stability and optical properties of MAPI films, and on the PCE and lifetime of the corresponding devices. FEFKFEFK peptide (F: phenylalanine, E: glutamic acid, K: lysine) was chosen due to its propensity to form β-sheets, and therefore a hydrogel.^28^ Additionally, FEFKFEFK contains lysine, which, as discussed previously, has been linked to improved perovskite moisture stability, as well as amino, carboxyl and aromatic functional groups which are known to passivate surface defects. We illustrate, through comparison of near-edge X-ray absorption fine structure (NEXAFS) and valence band (VB) spectra with density functional theory (DFT) predictions, that the peptide is incorporated into the MAPI film intact, and provide evidence that the peptide is homogeneously mixed with the perovskite. X-ray diffraction (XRD) patterns reveal that increasing the concentration of peptide leads to a decrease in MAPI crystallite size, attributed to the reduction in available hydrogel pore size.^28^ Absorption and photoluminescence (PL) spectra demonstrate a corresponding increase in bandgap, which is shown to mirror that expected due to quantum size effects. Optical measurements illustrate that the incorporation of peptide reduces the density of defect sites in the MAPI film, resulting in reduced nonradiative energy losses and boosting the device PCE compared with those based on pure MAPI. Peptide-incorporated MAPI devices are demonstrated to retain their improved PCE for longer in ambient conditions. The reasons for this enhanced stability are probed using X-ray photoelectron spectroscopy (XPS), in both ultrahigh vacuum (UHV) and (using near-ambient pressure XPS (NAP-XPS)), under 9 mbar water vapor. The latter corresponds to a realistic relative humidity (RH) of ca. 30%. We find a reduced loss of the organic (MA^+^) cation on heating peptide-incorporated materials both in UHV and in a moist environment. The latter is significant, as in previous studies of the incorporation of large organic molecules (such as ionic liquids^29^ or polymer hydrogels^12^), the passivating effect of the additive was found to be lost in the presence of water. We propose that the key difference here is that the additive is homogeneously incorporated into the composite material, forming a peptide network of small mesh size containing passivated MAPI crystallites, rather than giving rise to an additive-rich surface layer as previously observed.^12,29^ While the PCEs of the test devices reported here cannot rival those of fully optimized devices, this work demonstrates that peptide-encapsulated systems ultimately offer potential for water-stable OHPs with controllable pore size and hence tunable optical properties, making the optimization of such systems a productive avenue for further research.

## 2 Experimental Section

### 2.1 Materials

Indium tin oxide substrates (ITO), methylammonium iodide (MAI, 98%) and 2,2′,7,7′-tetrakis(N,N-di-*p*-methoxyphenylamine)-9,9′-spirobifluorene (Spiro-MeOTAD, 99%) were purchased from Ossila. Lead iodide (PbI_2_, 99%), anhydrous dimethylformamide (DMF, 99.8%), acetonitrile (ACN, 99.8%), chlorobenzene (99.9%),, 4-*tert*-butylpyridine (4-tBP, 98%), lithium bis(trifluoromethanesulfonyl)imide (LiTFSI, 99.95%), 4-*tert*-butylpyridine (4-tBP, 98%), lithium bis(trifluoromethanesulfonyl)imide (LiTFSI, 99.95%) and tris(2-(1H-pyrazol-1-yl)-4-*tert*-butylpyridine)-tris(bis(trifluoromethylsulfonyl)imide) (FK209, 98%) were purchased from Sigma-Aldrich. Tin(IV) oxide (SnO_2_) and Hellmanex III detergent were purchased from Alfa Aesar. FEFKFEFK peptide was synthesized by Biomatik.

### 2.2 Film and Device Fabrication

**Figure 1:**
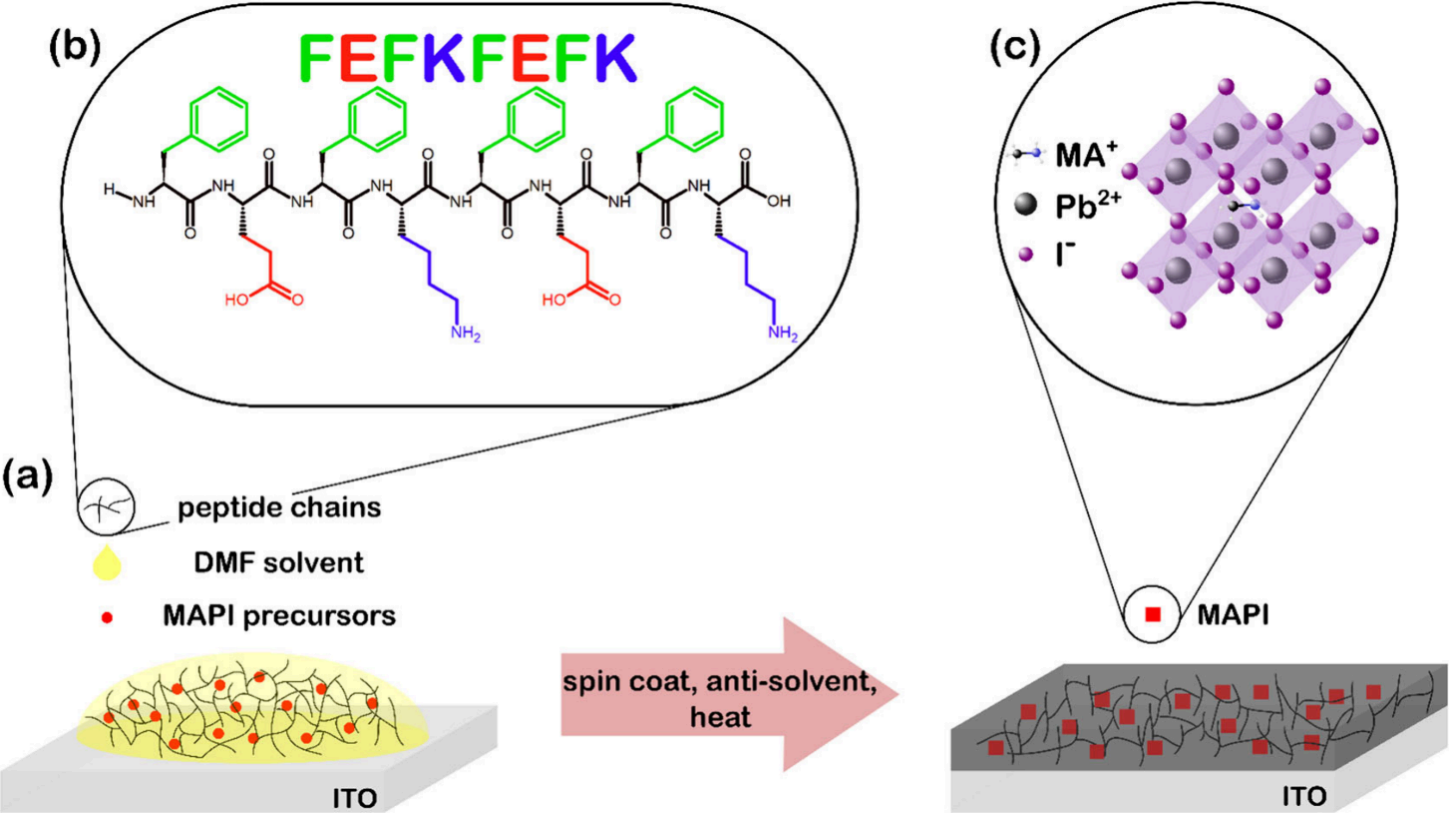
(a) Schematic illustration of the fabrication process for peptide-incorporated MAPI films. (b) Chemical formula of the peptide FEFKFEFK (F, phenylalanine; E, glutamic acid; K, lysine). (c) Crystal structure of MAPI.

To prepare the MAPI precursor solution, PbI_2_ (Alfa Aesar) was combined with DMF at a concentration of 1.1 M per mL and stirred at 70 °C for 1 h. Following cooling, MAI was added (1.1 M per mL) and the solution was further stirred under no heating for 30 min. FEFKFEFK was added at various concentrations to form a peptide hydrogel MAPI precursor solution. All peptide concentrations studied in this work were above the critical gelation concentration for FEFKFEFK, reported as 7 mg mL^–1^ (1.5 wt % corresponds to 10 mg mL^–1^).^30^ For the fabrication of MAPI thin films, unpatterned ITO glass substrates were cleaned via ultrasonication for 10 min in 3.0 vol % Hellmanex III diluted in deionized (DI) water, pure DI water, and ethanol sequentially. The substrates were dried using an air blower and then subjected to UV–O_3_ treatment for a further 15 min. Prior to deposition, substrates were heated to 70 °C. 100 μL of precursor solution was deposited onto the substrate and spin coated at 4000 rpm for 30 s. 200 μL ethyl acetate was added to the surface for the final 10 s of spin coating. The perovskite was then annealed at 100 °C for 10 min. This fabrication process is outlined in Figure 1.

For the fabrication of devices, patterned ITO glass substrates were cleaned via the process outlined previously. A 1 mL aliquot of SnO_2_ colloid dispersion (concentration: 15% in water) was diluted with 3 mL of deionized water, resulting in a transparent solution. This solution was subsequently deposited on an ITO substrate at a velocity of 3000 rpm for a duration of 30 s. Following the deposition, the coated substrate was subjected to an annealing process at a temperature of 150 °C for 1 h. The active MAPI layer was then deposited following the process outlined previously. After cooling, 70 μL of a solution containing 72.3 mg spiro-MeOTAD, 17.5 μL Li-TFSI in ACN (520 mg mL^–1^), 9 μL FK209 in ACN (300 mg mL^–1^) and 28.8 μL 4-tBP in 1 mL chlorobenzene, was spin coated at 2500 rpm for 30 s to form the hole transport layer (HTL). Finally, 100 nm Au was deposited by thermal evaporation under a vacuum as the cathode.

### 2.3 Characterization

Absorption spectra were measured using a Lambda 1050 ultraviolet–visible–near-infrared (UV–Vis–NIR) spectrometer (PerkinElmer). PL spectroscopy was carried out using a Fluorolog-QM spectrometer (Horiba), using a 450 nm excitation wavelength. The MAPI samples used for PL measurements were deposited on glass rather than ITO. Time-resolved PL was performed via time-correlated, single-photon counting (TCSPC) using a picosecond pulsed diode laser source with a wavelength of 450 nm. The detector was set to collect emitted photons at a wavelength of 770 nm, as determined from the static PL measurements. XRD analysis was performed on a D8 Discover (Bruker), using Cu Kα radiation. Device current–voltage (*J–V*) curves were measured using a Keithley 2420 source meter under AM 1.5G illumination (100 mW cm^–2^). Measurements were performed in ambient atmosphere and at room temperature (RT) without encapsulation. Scanning electron microscopy (SEM) was used to obtain the film morphology of the materials and was performed using a Zeiss Sigma VP instrument. TEM was used to obtain the bulk morphology of the films. Samples for TEM were prepared by simply spin coating either peptide, MAPI or MAPI peptide onto Cu grids followed by annealing and drying parameters, identical with those used for film deposition for the devices. TEM was performed using an FEI Talos F200A microscope.

### 2.4 XPS

Thermal degradation under UHV (<10^–8^ mbar) conditions was monitored using XPS (monochromatic Al kα, *h*ν = 1486.6 eV) the ESCA 2SR high throughput X-ray photoelectron spectrometer specifications and measurement details of which have been detailed elsewhere.^12^ Samples were measured at RT, 100 °C, 150 and 180 °C. All spectra were calibrated to the N 1s peak of MAPI (402.6 eV binding energy (BE)) due to the complexity of the C 1s region, because of the presence of the peptide. In the cases where no N peak was present, due to the degradation of the perovskite, the spectra were calibrated to the Pb 4f peak of metallic Pb (Pb^0^, 137 eV).^8,31^ BEs calibrated by both methods were found to be consistent in the cases where both chemical environments were present. XPS data were analyzed using CasaXPS software,^32^ in which a Shirley background and pseudo-Voigt peaks were fitted to core-level spectra. Surface stoichiometric ratios were calculated from the intensities of the fitted peaks, using relative sensitivity factors (RSFs) from the Scofield library. Errors on elemental compositions mainly arise from the setting of the background. In order to calculate the error on the peak areas, the upper and lower limits of what were deemed to be acceptable fits were taken and the atomic ratios obtained at these two limits were calculated.

### 2.5 NAP-XPS

Thermal degradation at 9 mbar water vapor pressure was measured using a laboratory-based NAP-XPS system, the specifications and measurement details of which can be found elsewhere.^12^ Samples were measured at RT using monochromatic Al kα X-rays (*h*ν = 1486.6 eV). Spectra were recorded under UHV conditions, under 9 mbar water vapor pressure at RT, 100 and 150 °C, and finally under UHV conditions after cooling to RT. Nine mbar corresponds to a relative humidity (RH) of ca. 30% at 25 °C. Spectra were calibrated and processed as outlined for the XPS measurements. RSFs for the analyzer are not known under NAP conditions, thus surface stoichiometric ratios were only calculated from the UHV measurements (i.e., before and after exposure).

### 2.6 Ultraviolet Photoelectron Spectroscopy (UPS)

UPS measurements were performed using a gas discharge vacuum ultraviolet (UV) source producing He I (21.2 eV) and He II (40.8 eV) radiation in the X-ray photoelectron spectrometer described in Section 2.4. In the case of the He I measurements, a bias voltage of −18.9 V was applied to the sample. Spectra were calibrated to the Fermi edge of Au. Errors on the VBM and SEED edge measurements were calculated from the linear fits to the edges. In the case of the SEED edges, this leads to a significant error as there are relatively few data points over the edge.

### 2.7 NEXAFS

NEXAFS measurements were performed at the Flexible PhotoElectron Spectroscopy (FlexPES) beamline, MAX IV Laboratory, Lund, Sweden, details of which can be found in our previous publications.^12,33^ For partial electron yield (PEY) C K-edge and N K-edge measurements, a 260 and 300 V cutoff was applied. Total electron yield (TEY) measurements, which are less surface sensitive, were measured via a drain current from the sample.

### 2.8 StoBe-deMon

Theoretical NEXAFS and VB spectra were produced using DFT calculations in the StoBe-deMon (ver. 3.3) software, for comparison with experimental results.^34^ Avogadro (ver. 1.2.0) was used to optimize the structure of individual amino acids, as well as FE and FK dipeptide chains, using molecular dynamics. The coordinates from the optimized structures were input into the StoBe-deMon package. C K-edge and N K-edge spectra were constructed by summing the spectra simulated for each individual carbon and nitrogen atom respectively for the individual amino acids which were then summed to give the peptide NEXAFS spectrum.^35^ Each spectrum was broadened using Gaussian functions with linearly increasing full-width at half maxima (fwhm) to account for the reduced lifetime of the σ* resonances.^36,37^ Due to limitations in the number of electrons that can be processed by StoBe-deMon, a simulated FEFKFEFK VB spectrum was constructed by summing the DFT-generated VBs for the dipeptides FE and FK, chosen to include the bonding interactions between F and E/K.

## 3 Results

### 3.1 NEXAFS

**Figure 2:**
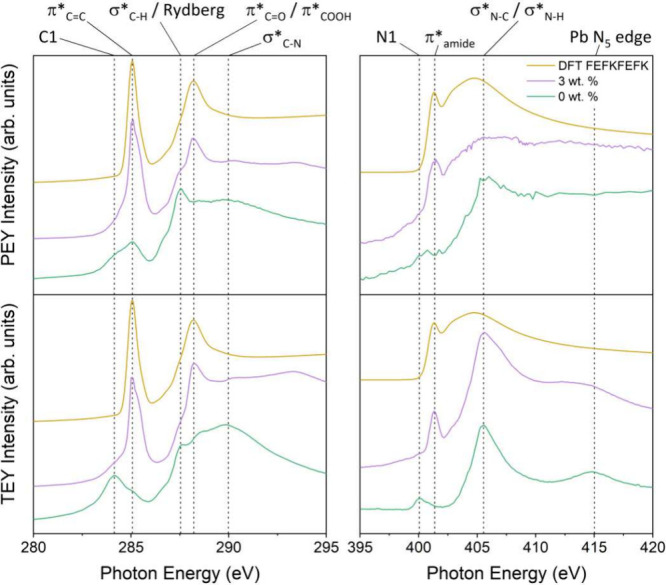
C K-edge (left) and N K-edge (right) PEY and TEY NEXAFS spectra from 0 and 3 wt % samples with prominent features labeled. A DFT simulated FEFKFEFK spectrum is included for comparison.

NEXAFS measurements were performed to identify resonant electronic transitions from core states to antibonding molecular orbitals. C K-edge and N K-edge spectra from pristine 0 and 3 wt % samples are displayed in Figure 2. Both TEY and PEY are displayed, providing more bulk and surface sensitive spectra, respectively. DFT simulated C K-edge and N K-edge spectra for the peptide are included.

The peaks at 287.5 and 290 eV in the C K-edge spectrum of the 0 wt % sample are attributed to the MA^+^ C–H and C–N σ* bonds, respectively.^38^ A broad feature between 283.5 and 286.0 eV (labeled C1) is also present in the 0 wt % spectrum, as found in our previous study.^12^ As argued previously, this feature is attributed to beam damage and adventitious carbon contamination.^38^ The C K-edge spectra of the 3 wt % sample also contains contributions from the peptide. The strong peak at 285 eV originates from the C=C double bonds in the aromatic ring of phenylalanine.^39^ Also present are features at 287.4 eV, attributed to both σ*_C–H_ and Rydberg excitations, 288.4 eV, associated with π*_C=O_ double bond in the peptide carboxyl group, and 288.5 eV, due to the π*_COOH_ transition in glutamic acid.^40,41^ The positions of these peptide-related peaks are in good agreement with the theoretical spectrum for the peptide.

The main N K-edge feature at 405.4 eV in the 0 wt % sample is a convolution of signals due to both σ*_N–C_ and σ*_N–H_ orbitals present in the perovskite organic cation.^42^ Also present in the 0 wt % spectrum is a resonance at 415 eV, previously reported as the Pb N_5_ edge.^42^ Both features remain prominent in the composite spectrum, which also contains a peak at 401.3 eV attributed to peptide bond π* transitions, in good agreement with the DFT spectrum of the peptide.^40,43^ The small peak at 400 eV (labeled N1) in the pure MAPI TEY spectrum has previously been reported as a consequence of beam damage or contamination.^44^

The presence of features originating from both MAPI and the functional groups of the peptide in the 3 wt % spectra confirms that following annealing and the formation of the MAPI film, the peptide remains intact as part of a mixed system. The relative intensity of MAPI and peptide features does not appear to differ substantially between TEY and PEY measurements, suggesting the composition at the surface is similar to the bulk, i.e. the peptide is distributed throughout the composite material.

### 3.2 Crystallographic, Optical, and Microscopy Characterization

**Figure 3:**
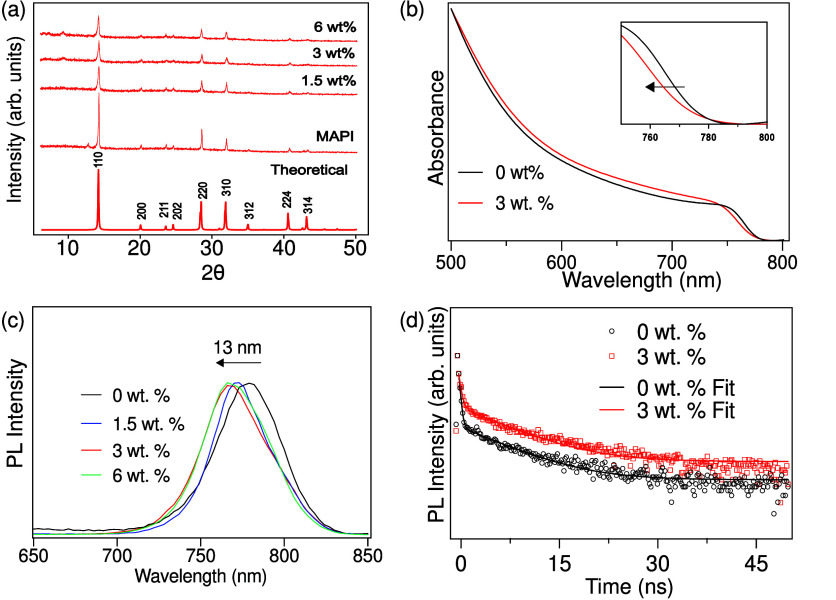
(a) XRD (patterns for higher peptide loadings are shown in Figure S1 of the Supporting Information). (b) UV–vis–NIR (spectra for higher loadings are shown in Figure S6). (c) PL spectra (normalized to peak height for clarity–unnormalized spectra are shown in Figure S5 of SI) of 0, 1.5, 3, and 6 wt % FEFKFEFK peptide. (d) Time-resolved PL spectra of MAPI containing 0 and 3 wt % peptide. Time-resolved PL spectra fitted with a double-exponential decay (0 wt %; see Supporting Information and Figure S5).

Figure 3a shows the XRD patterns of MAPI films containing 0 wt %, 1.5, 3, and 6 wt % FEFKFEFK. XRD patterns obtained for higher peptide loadings are shown in Figure S1 of the SI. All samples display characteristic peaks derived from MAPI, at 2θ of 14.1, 28.5, 31.9, and 35.0°, assigned to the (110), (220), (310), and (312) reflections, respectively. The PbI_2_ reflection at 12.7° is only present for the 0 wt % sample. This may be an indication that the inclusion of FEFKFEFK has enhanced the conversion of PbI_2_ into MAPI during fabrication or it has reduced degradation of the MAPI between fabrication and analysis. An increase in XRD peak width can be seen in the samples as the wt % of FEFKFEFK is increased, indicating that the peptide influences the MAPI crystal size. Table 1 displays the dependence of MAPI crystallite radius on peptide loading, estimated using the Scherrer equation for the (110), (220) and (314) XRD reflections. It is clear that crystal size decreases with peptide loading.

**Table 1:** MAPI Average Crystal Size Estimated from XRD Peak Width, Optical Gap Determined from UV–Vis–NIR Spectra, and Bandgap Estimated Using the Brus Equation Based on the Crystal Size (Details of the Parameters Used Are Given in the Supporting Information.)

sample	crystallite radius (nm)	measured optical gap (eV)	Brus bandgap (eV)
0 wt %	43 ± 4	1.58 ± 0.01	1.58 ± 0.01
1.5 wt %	27 ± 4	1.58 ± 0.01	1.58 ± 0.01
3 wt %	24 ± 4	1.59 ± 0.01	1.59 ± 0.01
6 wt %	22 ± 4	1.59 ± 0.01	1.59 ± 0.01
9 wt %	18 ± 2	1.60 ± 0.01	1.59 ± 0.01
12 wt %	17 ± 1	1.61 ± 0.01	1.59 ± 0.01
15 wt %	13 ± 2	1.62 ± 0.01	1.60 ± 0.01

**Figure 4:**
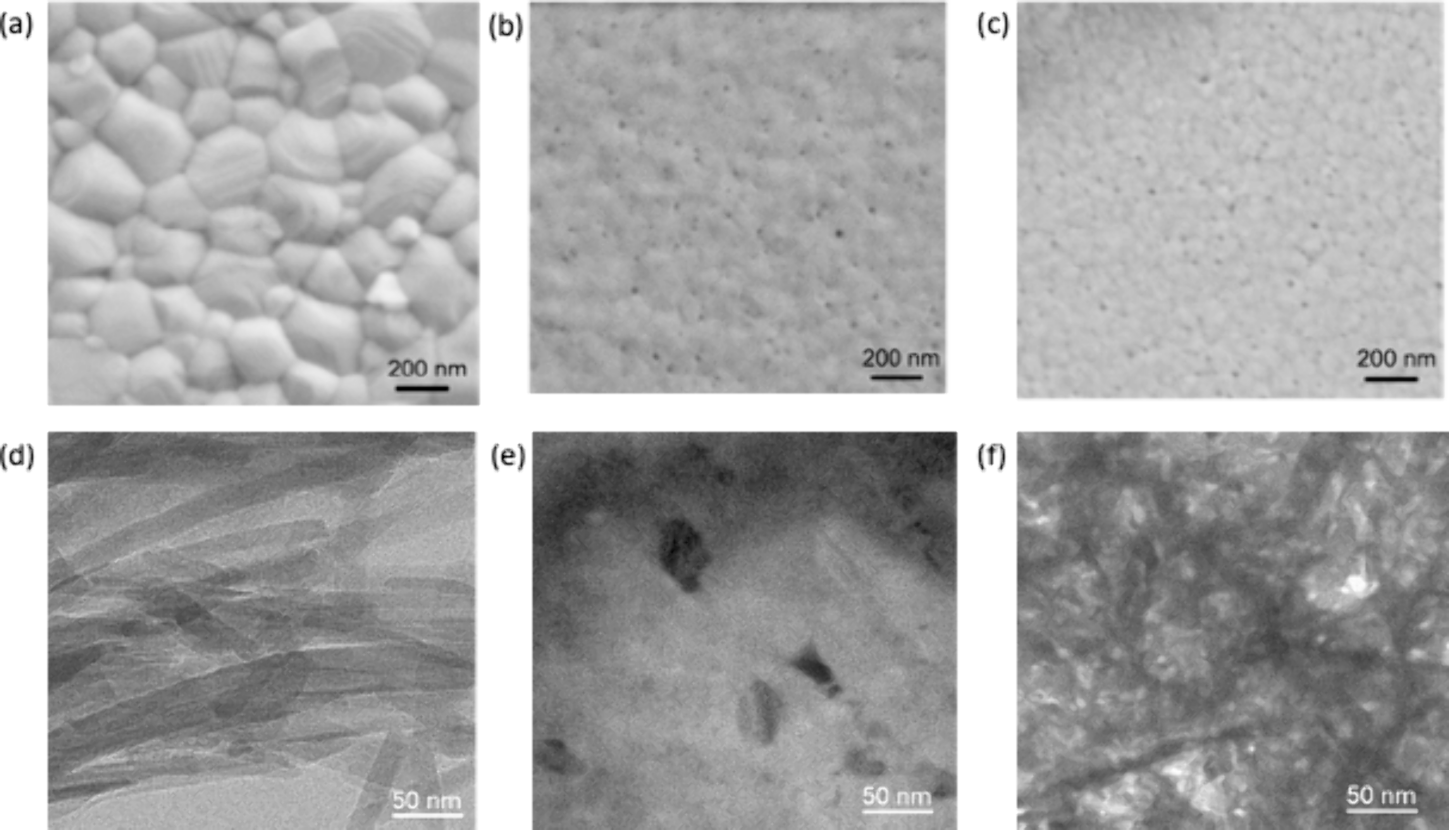
SEM images of (a) 0 wt % (b) 3 wt % (c) 6 wt %, showing a decrease in particle size as the peptide loading increases. Lower panels show TEM of (d) FEFKFEFK fibers, (e) 0 wt % and (f) 3 wt % samples. The peptide fibers can be seen as long criss-crossed structures with the peptide crystallites between them. Again, a decrease in MAPI crystallite size is observed in the image following addition of the peptide.

Fourier transform infrared (FTIR) analysis of the 6 wt % precursor solution, shown in Figure S2, confirms the formation of a peptide gel; hence, we attribute the reduction in particle size to the crystallization of MAPI within the pores of the peptide gel network (reported to have a size of 15–30 nm).^57^ At high loading, the increased density of peptide fibers results in a reduction in the effective “pore” size of the gel, and consequently appears to limit the crystal size. This decrease in particle size is supported by the SEM images in Figures 4 (a)- (c). A possible issue with the addition of the peptide is its relatively poor conductivity. The conductivity of the perovskite-peptide films decreased slightly in going from 0 to 6 wt % peptide loading (see Figure S3 and Table S1).

Rietveld refinement was applied to the diffraction patterns displayed in Figure S1, revealing a slight reduction in lattice parameter and cell volume with increased peptide loading, as shown in Figure S4. A similar effect was reported by Lang et al., and was attributed to the incorpo ration of lysine (substituting for two MA^+^ ions) within the crystal structure of MAPbBr_3_. However, the lattice parameter change observed here is an order of magnitude less than that reported for a similar lysine wt % by Lang et al.^17^ This suggests that there is very little incorporation of amino acid fragments within the crystal lattice of the perovskite.

Figure 3b shows the UV absorption spectra for MAPI samples with 0 and 3 wt % FEFKFEFK. A clear blue shift in the threshold edge that increases with peptide loading is seen, attributed to a slight increase in bandgap due to the decrease in crystal size evidenced by XRD. Table 1 displays the optical gaps for MAPI with various peptide concentrations determined from Tauc plots using the absorbance spectra (see SI Figure S6). The 0 wt % optical gap was found to be 1.58 ± 0.01 eV, in line with values from literature which lie between 1.55 and 1.6 eV.^31,45^

Also included in Table 1 are bandgaps estimated from the crystal radii displayed in Table 1 using the Brus equation (see eq S1 in Supporting Information), suggesting quantum size effects due to peptide-confined MAPI crystallization affect the bandgaps of the composite samples. These quantum confinement effects result from the limited crystallite size of the perovskite material as peptide loading increases. At the point where the exciton Bohr radius approaches the size of the nanoparticle it becomes spatially confined resulting in an increase in the energy of the band gap of the material.

Figure 3c displays PL spectra from each of the composite samples. As observed in the absorbance spectra, the photoluminescence emission exhibits a blue shift with increasing peptide loading, indicating an increase in bandgap due to the inclusion of peptide. It is also evident that PL intensity increases logarithmically with peptide loading, as demonstrated in Figure S5, suggesting that the inclusion of the peptide helps reduce the density of defect sites, thus diminishing nonradiative recombination pathways.

Figure 3d displays the photoluminescence lifetime of two peptide loadings (0 and 3 wt %), fitted using a double-exponential decay model (see eq S2).^46^ The fluorescence lifetimes were determined from the model parameters using eq S3 and are displayed in Table 2, clearly showing an increase in PL lifetime with peptide loading, again suggesting the removal of rapid recombination processes at defect sites. As shown in Figure S4, at higher loading (6 wt %) a monoexponential decay function fits the decay well, whereas the PL lifetime decay of the 0, 1.5, and 3 wt % samples cannot be explained by such a model, further implying that an increased peptide loading reduces nonradiative energy loss caused by defect sites.^47^

**Table 2:** Fluorescence Lifetimes Calculated from Time Resolved PL Measurements^a^

Sample	Lifetime (ns)
0 wt %	1.19 ± 0.06
1.5 wt %	1.39 ± 0.31
3 wt %	2.00 ± 0.10
6 wt %	6.00 ± 0.10

a See Supplementary Information eqs 2–4 and Figure S6 for details of the calculations.

Figure 4 shows SEM images of MAPI with peptide loadings of 0, 3, and 6 wt %. In agreement with the XRD findings the particle size is found to decrease with peptide loading. TEM images shown in Figures 4(d) – (f) show the pure peptide, pure MAPI perovskite (0 wt %) and MAPI with 3 wt % peptide respectively. In the TEM image of the pure peptide the fibrous nature of the peptide can be observed, which is absent in the TEM of the pure MAPI (Figure 4(e)). The peptide-MAPI mixture however shows evidence of the peptide fibers, with high contrast, and crystalline structures between the fibers. It appears from these images that the peptide may act as nucleation sites for crystallization of the MAPI, which leads to the high contrast of the fibers in the image, since the pure peptide, as expected, shows a lower contrast than the pure MAPI, as seen in Figures 4(d) and (e).

### 3.3 Valence Band and Work Function

UPS spectra of the 0 and 3 wt % samples were obtained using both He I (21.2 eV) and He II (40.8 eV) radiation to provide further insight into the electronic structure of the peptide composite.

**Figure 5:**
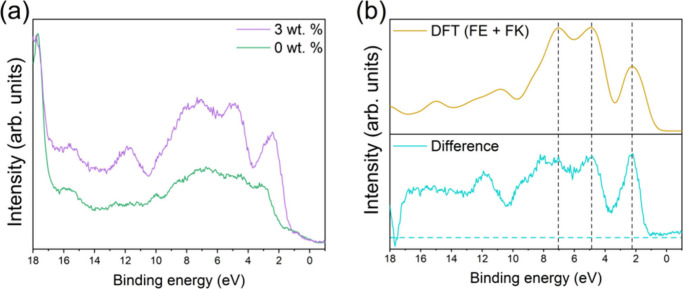
(a) VB spectra obtained using a He II UV source. (b) VB difference spectrum calculated by subtracting at 0 wt % VB from the 3 wt % VB (bottom) and a DFT simulated VB for FE+FK for comparison (top).

VB spectra from both samples, obtained using the He II source, are displayed in Figure 5a. Figure 5b shows the VB difference spectrum, produced by normalizing and aligning both VB spectra to the strong Pb 5d_5/2_ core level peak from MAPI at 17.7 eV BE, before subtracting the 0 wt % spectrum from the 3 wt % spectrum.^48^ Also included in Figure 5b is a simulated VB spectrum for the peptide, estimated by summing the DFT-simulated VBs of FE and FK. The experimental difference spectrum exhibits a similar shape to the simulated peptide VB, with strong features at 2.2 and 4.8 eV BE. VB features at these BEs have previously been reported in other benzene ring-containing systems; thus, we assign these peaks, at least in part, to the benzyl side chain of phenylalanine.^49−51^ Adsorbed atmospheric contaminants, such as H_2_O, CO_2_ and CO, are known to produce strong peaks at both ca. 5.0 and 9.0 eV; however, any features from such species are expected to be significantly reduced in intensity in the VB difference spectrum.^52^ The presence of contributions from the peptide in the VB spectra displayed in Figure 4 support our observations from NEXAFS that the peptide is incorporated into the MAPI film intact.

**Figure 6:**
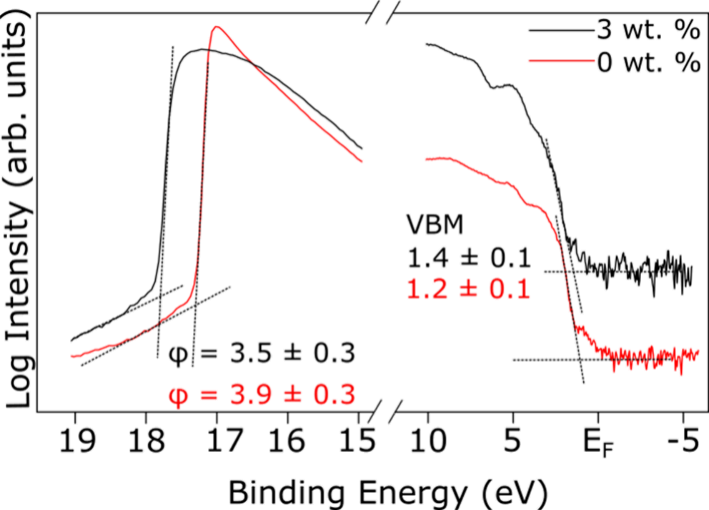
SEED edge (left) and VB (right) spectra of 0 and 3 wt % samples measured using a He I UV source (*h*ν = 21.2 eV). The black dashed lines indicate the linear fits used to determine the work functions and VBM of the samples.

The VB edge and secondary electron energy distribution (SEED) edge regions, measured using the He I source, are displayed in Figure 6. The valence band maximum (VBM) and work function (φ) of each sample was determined by applying a linear fit to the leading edge and baseline of both the VB and SEED and extrapolating to the point of intersection. A logarithmic intensity scale was employed as previous reports indicate that an unusually low density of states (DOS) at the VBM of organic–inorganic lead halide perovskites may lead to inaccurate VBM estimations when determined from linear intensity plots.^53^ The work function of the samples was obtained using eq 3:
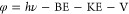
3By accounting for the applied bias, V, employed to sharpen the SEED edge by providing 0 (zero) kinetic energy (KE) photoelectrons with enough energy to reach the detector, and aligning the spectrum to the Fermi energy, the work function φ can be obtained by subtracting the binding energy cut off of the SEED edge from the photon energy, as shown in Figure 6. The VBM of the pure MAPI sample is calculated to lie at a BE of 1.2 ± 0.1 eV, in good agreement with previous studies of MAPI which report values between 1.2 and 1.4 eV.^53,54^ The 3 wt % sample also exhibits a VBM BE of 1.2 ± 0.1 eV, indicating that the inclusion of peptide has no effect on the VBM of the sample. This is consistent with Figure 5b, where it can be seen that the peptide makes no contribution to the valence band density of states below 1 eV BE. As discussed in Section 3.2, the bandgap of the 0 wt % sample is 1.58 ± 0.01 eV, indicating that the Fermi energy of MAPI is closer to the conduction band than the valence band, meaning the material is n-type in character, as expected.^31,45^ As the change in bandgap due to the inclusion of the peptide is small, as shown in Table 2, the 3 wt % sample is also an n-type semiconductor. From the VBM and SEED edge the work function of the 0 wt % sample is found to be 3.9 ± 0.3 eV, within error of the 4.0 eV previously reported for MAPI.^53^ The 3 wt % peptide sample has a work function of 3.5 ± 0.3 eV, showing a small reduction from the value for MAPI.

### 3.4 Device Performance

**Figure 7:**
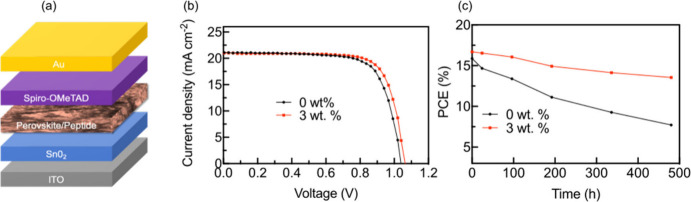
(a) Schematic diagram of the PSC device architecture. (b) *J*–*V* curve of PSCs with active layers containing 0 and 3 wt % peptide. (c) Evolution with time of PCE of 0 and 3 wt % devices stored at 35% RH.

To determine the effect of the peptide on photovoltaic performance, PSCs based on composites containing various concentrations of peptide were produced. Thin-film devices were built with the architecture shown in Figure 7a. A maximum PCE of 16.8% was achieved with a peptide loading of 3 wt %, compared with 15.9% from a 0 wt % device. This slight increase is likely to be due to a reduction in defect sites, evidenced by the data reported in section 3.2. Photovoltaic parameters of PSCs at these two peptide loadings calculated from the J-V curves displayed in Figure 7b are listed in Table 3. The 3 wt % device exhibited a small increase in short circuit current density (*J*_SC_) and open circuit voltage (*V*_OC_) compared to the 0 wt % device, linked to the increase in PL intensity and charge carrier lifetimes displayed by the peptide composites (see Figures S3 and 3d). Fill-factor (FF) is also improved by the inclusion of peptide, probably due to the reduction of defect trap states reported in section 3.2, resulting in enhanced charge carrier mobility and reduced recombination, both of which have a strong influence on FF.^55−58^ It should be noted that at peptide concentrations greater than 3 wt %, a decrease in PCE was observed (see Supporting Information Figure S8), likely to be due to the formation of peptide-rich areas at grain boundaries negatively impacting charge transfer due to the poor conductivity of FEFKFEFK. As noted in section 3.2, a small reduction in conductivity is observed as a function of peptide loading (Figure S3).

**Table 3:** Photovoltaic Parameters of PSCs with 0 and 3 wt % Peptide^a^

sample		*J*_SC_ (mA cm^–2^)	*V*_OC_ (V)	FF (%)	PCE (%)
0 wt %	average	21.0 ± 0.2	1.03 ± 0.01	72.1 ± 1.6	15.6 ± 0.4
	champion	21.1	1.04	72.5	15.9
3 wt %	average	20.9 ± 0.1	1.06 ± 0.01	74.7 ± 0.6	16.6 ± 0.1
	champion	21.0	1.06	75.1	16.8

a Average parameters were calculated from a set of 8 devices.

To determine the long-term stability of these PSCs, devices were stored at RT in 35% RH and the evolution of PCE was monitored with time, as shown in Figure 7c. The 3 wt % device maintained 81% of its maximum PCE after 480 h, whereas the 0 wt % device maintained only 48% over the same period. This suggests that the inclusion of peptide enhances the stability of MAPI in ambient conditions.

### 3.5 Thermal Decomposition under UHV Conditions

**Figure 8:**
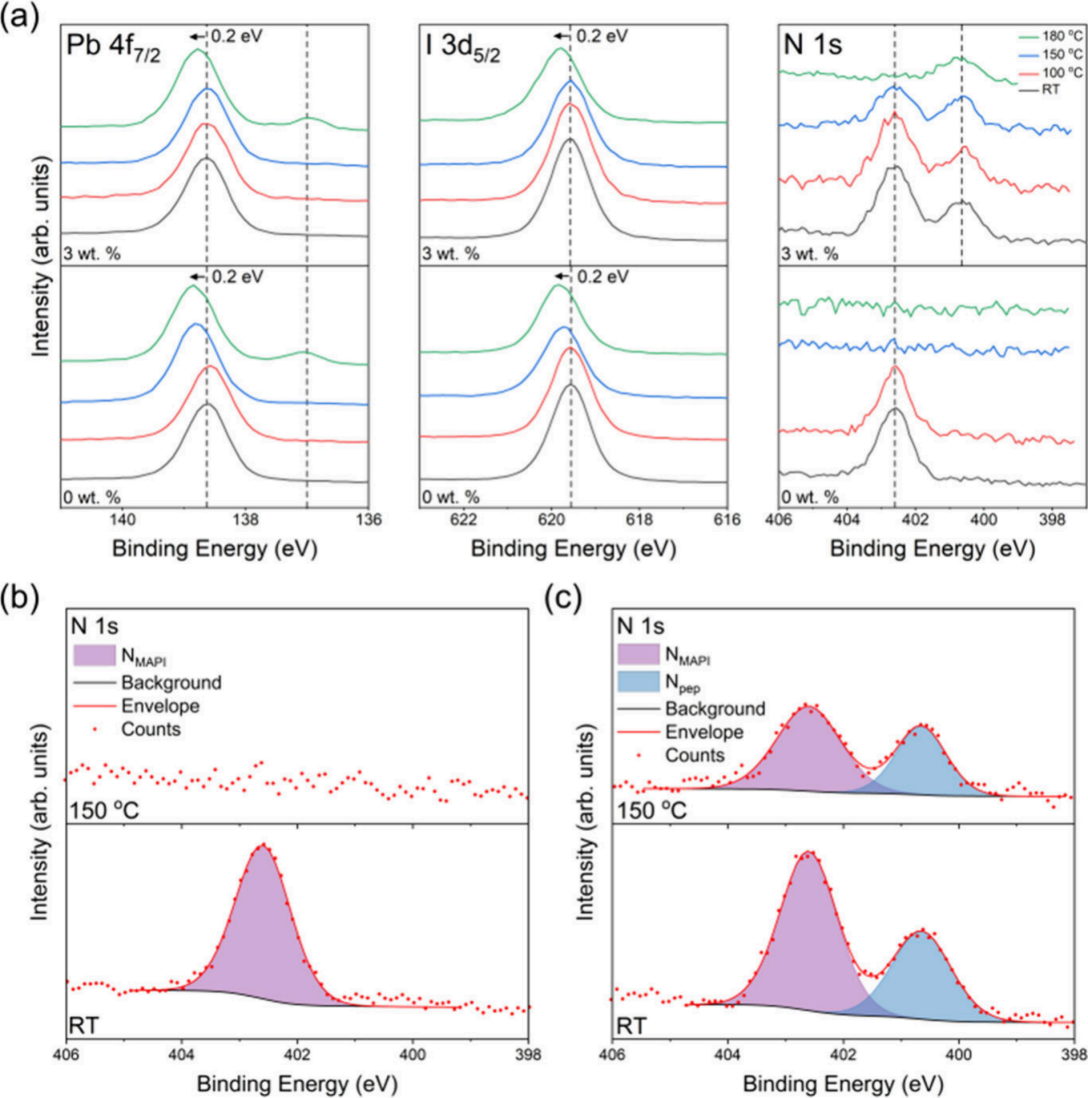
(a) Pb 4f_7/2_, I 3d_5/2_, and N 1s high-resolution core-level XPS spectra of 0 and 3 wt % samples, measured in UHV at RT, 100 °C, 150 °C, and 180 °C. Initial peak positions are indicated by the dashed lines, and the final shift is noted (as measured following cooling). (b) 0 wt % and (c) 3 wt % N 1s core level spectra at RT and 150 °C with fitted peaks labeled.

A comparison was made of the surface chemistries of the 3 wt % composite (with optimum PCE) and the pure perovskite. The reduction in conductivity of the samples as a function of loading (Figure S3) limited XPS data collection at higher peptide loadings. To discriminate between temperature- and moisture-induced changes to surface chemistry, MAPI-peptide samples were first heated under UHV conditions. High-resolution core level XPS spectra were acquired once a constant temperature was achieved at RT, 100 °C, 150 °C, 180 °C and after cooling. Pb 4f_7/2_, I 3d_5/2_ and N 1s core level spectra are displayed in Figure 8a.

At RT, the MAPI Pb^2+^ Pb 4f_7/2_ peak appears at 138.6 ± 0.1 eV BE for both samples, in excellent agreement with previous studies of MAPI.^8,31^ The decomposition of MAPI to PbI_2_ can be identified in XPS by a BE shift of +0.2 eV.^59^ This is seen to occur at 150 °C in the 0 wt % and 180 °C in the 3 wt % spectra, suggesting the inclusion of the peptide has led to an improvement in thermal stability.^31^ A smaller peak at 137.0 ± 0.1 eV becomes more prominent at 180 °C in both samples and is assigned to Pb^0^, a product of thermal or X-ray induced decomposition of PbI_2_.^31,60^ A single I 3d_5/2_ peak at 619.6 ± 0.1 eV BE is present at RT for both samples, in line with previous studies of MAPI.^8,31^ A BE shift of +0.2 eV occurs at 150 °C for the 0 wt % and 180 °C for the 3 wt % samples, consistent with the degradation of MAPI to PbI_2_ and in agreement with the observations from the Pb 4f spectra.

The RT N 1s spectra of both samples exhibit a peak at 402.6 ± 0.1 eV BE, which as mentioned above arises from the MA^+^ cation in MAPI and is used to reference the BE scale.^8^ A second peak at 400.7 ± 0.1 eV BE in the 3 wt % spectra is assigned to the amide bond in the peptide, also found to be present in the N 1s spectrum of FEFKFEFK deposited on ITO shown in Figure S5.^61−63^ During heating, the 402.6 eV peak is completely lost at 150 °C for the 0 wt % pure MAPI and 180 °C for 3 wt % peptide-loaded sample. This indicates the complete loss of nitrogen from the perovskite in the surface region in the form of ammonia gas during the decomposition of MAPI to PbI_2_, in line with the process outlined in eq 2.^8,64^ The decomposition of the organic cation occurs at a higher temperature for the 3 wt % sample, consistent with the temperatures at which BE shifts are observed in the Pb 4f_7/2_ and I 3d_5/2_ spectra. Enlarged N 1s regions for the 0 and 3 wt % samples are displayed in Figures 8b and 8c respectively, highlighting the loss of surface MA^+^ nitrogen at 150 °C in the 0 wt % sample, in contrast to the 3 wt % sample where significant surface MA^+^ remains.

**Figure 9:**
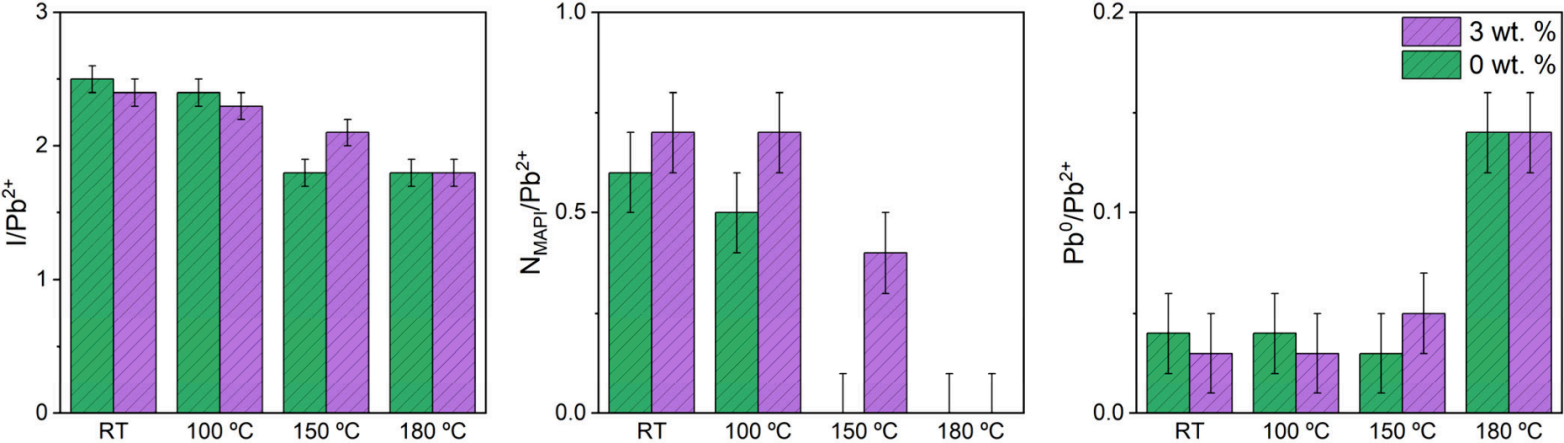
Surface stoichiometry of 0 and 3 wt % samples as a function of temperature, calculated from the areas of fitted XPS peaks. I, N_MAPI_, and Pb^0^ concentrations with respect to Pb^2+^ are shown. Values are listed in Tables S2 and S3. The nominal stoichiometry of MAPI is 1:3:1 (Pb^2+^/I/N).

Elemental concentrations, calculated from peak areas fitted to high-resolution core level spectra, are shown in Figure 9. The I/Pb^2+^ ratios at RT were found to be 2.5 ± 0.1 and 2.4 ± 0.1 for the as-prepared 0 and 3 wt % samples, respectively. The slight discrepancy with the nominal value for MAPI of 3 is likely to be due to degradation between fabrication and analysis. These values reduce to 1.8 ± 0.1 and 2.1 ± 0.1 at 150 °C for the 0 and 3 wt % samples respectively, and 1.8 ± 0.1 for both samples at 180 °C. This implies improved stability of the 3 wt % sample at 150 °C, consistent with the observed shifts in peak BEs due to the formation of PbI_2_ in Figure 8a. N_MAPI_/Pb^2+^ ratios at RT were found to be 0.6 ± 0.1 and 0.7 ± 0.1 for the 0 and 3 wt % samples, respectively. Again, this is lower than the nominal value of 1 for MAPI, due to some degradation before analysis. At 150 °C for the 0 wt % and 180 °C for the 3 wt % sample, the N signal from MA^+^ is completely removed. A similar concentration of Pb^0^ was displayed by both samples, increasing from 0.04 ± 0.01 and 0.03 ± 0.01 at RT for the 0 and 3 wt % samples respectively, to 0.14 ± 0.01 at 180 °C. The shifts in the binding energies of the peaks, observed in the core level XPS spectra, and the changes in the I and N_MAPI_ concentrations indicate that the inclusion of peptide raises the temperature threshold for the thermal degradation of MAPI.

The N 1s peak at 400.7 eV BE assigned to the peptide bond present in the 3 wt % core level spectra remains after heating to 180 °C. The evolution of peptide concentration determined from this peak (N_pep_) is shown in Table S3. The N_pep_/Pb^2+^ ratio in the 3 wt % sample reduces from 0.4 ± 0.1 at RT to 0.2 ± 0.1 at 180 °C, indicating that there is some loss of peptide during heating. The nominal N_pep_/Pb^2+^ ratio for the 3 wt % sample was calculated as 0.2, indicating that the surface composition does not differ strongly from the bulk. Thus, the majority of the peptide is incorporated within the MAPI film, rather than existing as a layer at the surface. This is in contrast to our previous studies of polymer hydrogel perovskite additives, where a surface additive concentration ca. 10 times the bulk concentration was observed,^12^ or ionic liquid additives, where a similar surface enhancement was measured.^29^ It should also be noted that no difference in inelastic background shape is evident in the survey spectra of these samples (see Figure S6), whereas in our previous studies of perovskite additives, the inelastic scattering of photoelectrons by the overlayer results in a clear shoulder to the higher BE side of core level peaks.^12^ The homogeneous mixing of the peptide gel in the perovskite film is also suggested by the C and O concentrations calculated from the C 1s and O 1s spectra at RT displayed in Figure S7. The changes to the C 1s spectrum when 3 wt % peptide is added are consistent with the spectrum of FEFKFEFK in Figure S5. The Pb^2+^:C:O stoichiometry of the 0 wt % film was found to be 1 ± 0.1:2.3 ± 0.1:1.0 ± 0.1, differing from the nominal values of 1:1:0 due to surface carbon and oxygen contamination. That of the 3 wt % stoichiometry was found to be 1 ± 0.1:5.7 ± 0.1:1.4 ± 0.1, not substantially different from the nominal values of 1:1.9:0.2, when a similar amount of adventitious contamination is allowed for. These values are in contrast to our previous study of polymer hydrogel additives, which displayed C and O concentrations significantly higher than expected.^12^ These observations are consistent with crystallization of MAPI within the pores of the peptide gel network, as suggested by the XRD results.

### 3.6 Thermal Decomposition at 9 mbar Water Vapor Pressure

**Figure 10:**
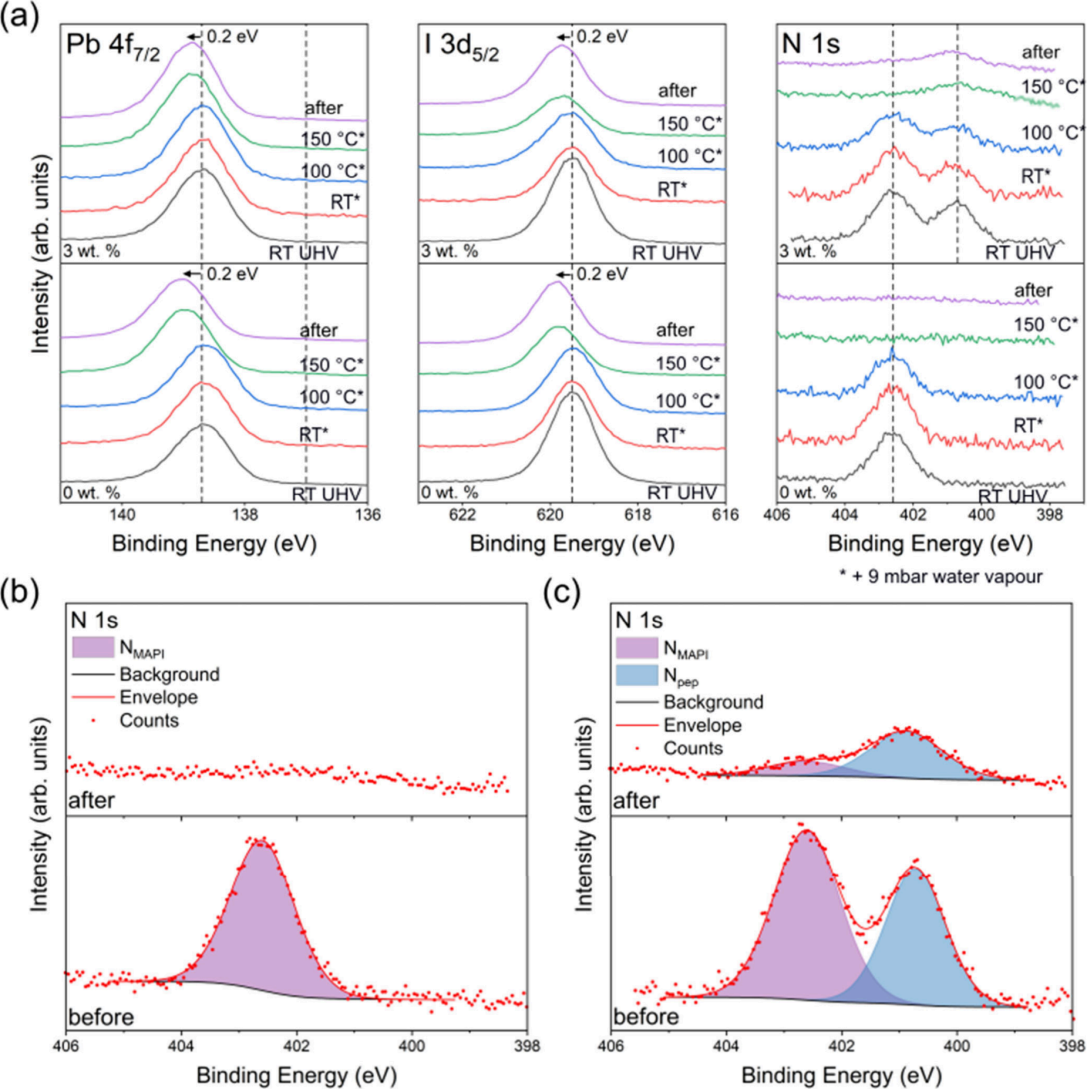
(a) Pb 4f_7/2_, I 3d_5/2_, and N 1s high-resolution core-level XPS spectra of 0 and 3 wt % samples, measured under UHV conditions at RT; then at RT, 100, and 150 °C under 9 mbar water vapor pressure; and finally under UHV following cooling to RT. Initial peak positions are indicated by the dashed lines, and the final shift is noted (as measured following cooling). (b) 0 wt % and (c) 3 wt % N 1s core level spectra before and after the heating regime at 9 mbar water vapor pressure, with fitted peaks labeled.

To determine the influence of moisture at various temperatures on the MAPI and MAPI-peptide films, high-resolution core level XPS spectra were acquired during water vapor exposure using NAP-XPS. Samples were measured under UHV at RT (UHV before), then at RT, 100 and 150 °C in the presence of 9 mbar water vapor (ca. 30% RH), and finally at UHV after cooling to RT and the removal of water vapor (UHV after). Pb 4f_7/2_, I 3d_5/2_ and N 1s core level spectra recorded during this heating regime are displayed in Figure 10a.

Consistent with our UHV study, the Pb 4f_7/2_ spectra for both samples initially consist of a single Pb^2+^ feature at 138.7 ± 0.1 eV BE. A BE shift of +0.2 eV is observed under 9 mbar at 150 °C for both samples, suggesting that, unlike the UHV XPS study, the samples begin to degrade at same stage. For both samples, the MAPI I 3d peak under 9 mbar at 150 °C shifts from an initial BE of 619.5 ± 0.1 eV to 619.7 ± 0.1 eV, consistent with the shift observed in the Pb 4f spectra. The N 1s MA^+^ peak at 402.6 ± 0.1 eV BE disappears under 9 mbar at 150 °C in both samples; however, following the removal of water vapor, a small feature is clearly distinguishable at 402.6 eV ± 0.1 eV BE in the 3 wt % N 1s spectra, suggesting some MA^+^ remains at the surface of the 3 wt % sample following the heating regime at 9 mbar water vapor. Enlarged N 1s regions before and after the heating regime at 9 mbar water vapor pressure are shown in Figures 10b and 10c for the 0 and 3 wt % samples respectively, demonstrating the retention of some MA^+^ at the surface of the latter. These regions are also compared with results obtained after heating in UHV in Figure S8. The N_pep_ peak at 400.7 eV ± 0.1 eV BE assigned to the amide bond in the peptide is present in the N 1s spectra, and remains, with attenuated intensity, after heating to 150 °C, consistent with the UHV XPS results discussed above.

Elemental concentrations were determined from fitted peak areas from the spectra recorded in UHV, before and after heating under 9 mbar water vapor pressure. An I/Pb^2+^ ratio of 2.4 ± 0.1 was calculated for both samples, but this was reduced to 1.6 ± 0.1 and 1.7 ± 0.1 following heating for the 0 and 3 wt % peptide samples, respectively. An initial N_MAPI_/Pb^2+^ ratio of 0.7 ± 0.1 and 0.9 ± 0.1 was measured for the 0 and 3 wt % samples, respectively. This value reduced to zero in the 0 wt % sample, but a trace (N_MAPI_/Pb^2+^ = 0.1 ± 0.1) remained following heating in the 3 wt % peptide sample. The survival of some MA^+^ at the surface following the heating regime at 9 mbar water vapor pressure indicates that the incorporation of peptide leads to a slight improvement in thermal stability in the presence of moisture. A Pb^0^/Pb^2+^ ratio of 0.02 ± 0.01 in pure MAPI and 0.01 ± 0.01 for the 3 wt % peptide sample was found following heating in water. In line with the UHV XPS results, the N_pep_/Pb^2+^ ratio fell by a similar fraction, from 0.7 ± 0.1 to 0.3 ± 0.1 in the 3 wt % sample, suggesting loss of peptide during heating in 9 mbar water.

## 4 Discussion

The homogeneous incorporation of the peptide into the perovskite composite is evidenced in our experimental data in a number of ways. The strong similarity between the 3 wt % TEY and PEY NEXAFS spectra demonstrates that the surface composition of the peptide composite is similar to that of the bulk. Likewise, quantification of surface N, O and C from XPS reveals no evidence for a thick additive-rich overlayer in the 3 wt % sample, as N, C and O concentrations differ little from calculated nominal values for a homogeneously mixed system. Similarly, the inelastic background observed in XPS is characteristic of a homogeneously mixed system.^65^ This contrasts with our previous studies of polymer hydrogel and ionic liquid additives, where typically a thick (several nm) additive rich surface overlayer is observed.^12,29^ Further to this, the presence of NEXAFS and VB features predicted by DFT simulations of the FEFKFEFK molecule provides evidence that the peptide is incorporated into the MAPI film intact, while FTIR confirms the formation of a peptide gel. This evidence of homogeneous mixing of peptide and MAPI, coupled with the formation of a gel, may suggest that crystallization of the perovskite occurs within the pores of the peptide gel network. This is also evidenced by the TEM (Figure 4(f)) showing the perovskite particles embedded in the peptide network. Within this model of incorporation, the broadening of XRD peaks with increased peptide loading, and the corresponding decrease in crystallite size shown in Table 1, can be explained by the reduction in available pore size as the concentration of peptide is increased.^28^ These observations appear to be corroborated by the blue shifts in UV absorption and PL emission, and the corresponding increase in bandgap, which (given the simplicity of the model) agrees well with that predicted on the basis the quantum size effects arising from the confined crystallite size. Rietveld refinement of the XRD patterns shows that there is very little change in the MAPI lattice parameters with peptide loading. Thus, while we cannot rule out the incorporation of a very small amount of amino acid fragments within the MAPI crystal structure itself, it is clear that the reduction in crystallite size is the primary factor influencing the change in bandgap.^17^

The optimal loading for device performance was found to be 3 wt % (corresponding to 20 mg mL^–1^, greater than the critical gelation concentration of 7 mg mL^–1^)^30^ with the peptide composite PSC exhibiting a maximum PCE of 16.6%, compared with 15.9% from a 0 wt % device. This improvement in efficiency is attributed primarily to a reduction in defect sites, evidenced by the data displayed in Figures 3, and S5. This enhancement due to the inclusion of the peptide offsets the negative impact on charge transfer due to the poor electrical conductivity of FEFKFEFK, which has a greater impact on device performance at higher loading (and also limited the collection of XPS data at loadings higher than 3 wt %). The 3 wt % device was also demonstrated to maintain 81% of its maximum PCE following 480 h of storage in 35% RH, in contrast to 48% achieved with a 0 wt % device. This improvement in device durability demonstrates an improvement in material stability with the inclusion of peptide, and is most likely to be due to a reduction in the formation of nonradiative energy loss mechanisms due to atmospheric degradation.

**Figure 11:**
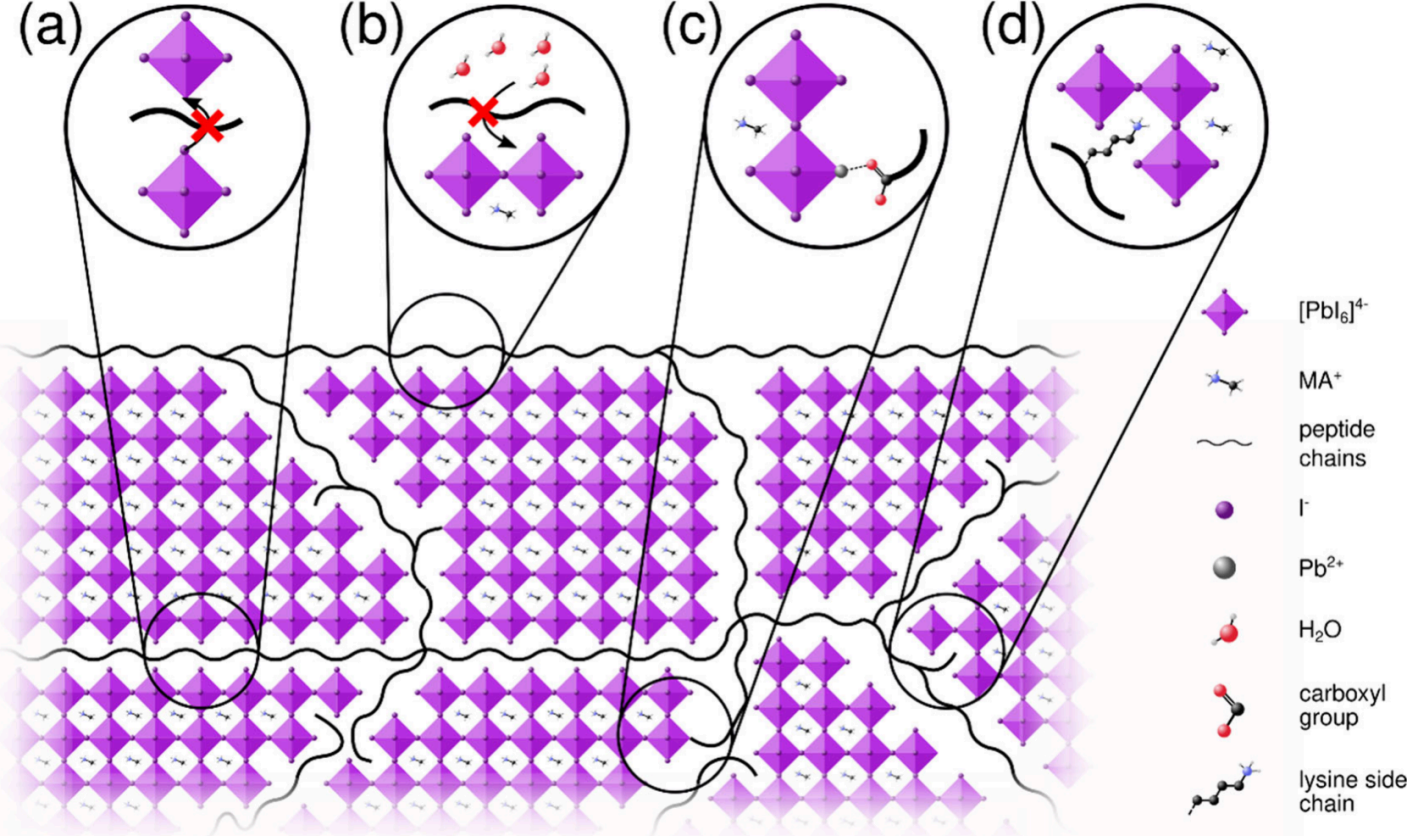
Schematic diagram of peptide-incorporated MAPI, showing MAPI crystallites encapsulated by a network of peptide chains. Proposed mechanisms for enhanced stability are included: (a) inhibited ion migration, (b) reduced water ingress, (c) passivation of surface defects by peptide termini (in this case an undercoordinated Pb^2+^ is passivated by a carboxyl anion terminus^41−43^), and (d) passivation of surface defects by amino acid functional groups (in this case an MA^+^ vacancy is passivated by a protonated lysine side chain^37^).

The comparative *in situ* thermal stress XPS studies of these composites, at both UHV and 9 mbar water vapor pressure, demonstrate at the molecular level the reasons for the enhancement in material stability provided by the peptide gel. Under heating, the degradation of the perovskite is initiated by the loss of surface I, maintained by the migration of I^–^ ions via vacancies in the perovskite lattice, and the decomposition of the organic cation, resulting in the loss of surface N in the form of ammonia gas.^29,60^ Thus, the evolution of I and N concentration with heating provides a clear picture of the degradation of MAPI. Stoichiometric analysis of the 0 wt % sample reveals that the MAPI undergoes significant decomposition at 150 °C, evidenced by a marked drop in I/Pb^2+^ and N_MAPI_/Pb^2+^ ratios shown in Figure 8 and Table S2. A similar reduction occurs in the 3 wt % sample, but only when heated to 180 °C, shown in Figure 8 and Table S3. This demonstrates that the inclusion of the peptide raises the threshold for thermally induced degradation of the perovskite. It is likely that the encapsulation of the MAPI crystals by the peptide network, evidenced by our characterization measurements, inhibits both the migration of ions and ingress of water vapor, thus slowing the rate of decomposition. NAP-XPS measurements at 9 mbar water vapor pressure demonstrate that some of this enhanced stability is retained in the presence of moisture, with trace N_MAPI_ remaining at the surface of the 3 wt % sample following the heating regime. This is in contrast to other additives studied at near-ambient pressure, such as some ionic liquids, which completely lose their advantage over pure perovskite in atmospheric conditions.^29^ The key difference here appears to be that the additive is homogeneously incorporated into the composite material, forming a hydrogel network of small pores containing passivated MAPI crystallites, rather as a thick additive-rich hydrogel surface layer.^12,29^ This difference may be associated with greater availability of functional sites in the peptide compared with pHEMA. While pHEMA contains only C=O and OH groups, which coordinate strongly with positively charged defects, the peptide studied here contains functional groups that are associated with passivating positive, negative and neutral defects in perovskites.^14,16−21,23,24^Figure 11 demonstrates the proposed mode of peptide incorporation within the MAPI film, and shows likely models for the enhanced stability and surface passivation evidenced by our XPS and optical measurements. The hydrogel provides an ideal environment both for facile vacancy passivation by the peptide side chains (for example the substitution of a protonated lysine unit of the peptide for a missing MA^+^ ion^37^) or for ligand binding to otherwise undercoordinated surface ions.^20,21^

The performance enhancement observed here is modest, and clearly significant optimization of such systems is necessary to improve the efficiency and stability of peptide-passivated systems. However, our work demonstrates that this is a productive avenue of research, as these systems offer the potential for a more sustainable route to water-stable PSCs with controllable pore size and hence tunable optical properties.

## 5 Conclusions

We have investigated the crystallographic and optical properties of peptide-hydrogel-incorporated MAPI perovskite films and studied their surface degradation as a function of temperature and water vapor pressure. It is shown that the inclusion of FEFKFEFK peptide in the perovskite precursor solution results in MAPI crystallization within the pores of the hydrogel, giving rise to an increase in band gap attributed to quantum size effects. The MAPI crystallite size is shown to decrease with increasing peptide concentration, corresponding to a decrease in available pore size.^28^ Optical measurements demonstrate that the resulting MAPI-peptide films exhibit reduced nonradiative recombination pathways, attributed to the passivation of defect sites and undercoordinated surface ions by the peptide. Reduced nonradiative losses are also evidenced by the enhancement in device PCE, which was optimized to 16.8% at 3 wt %, compared with 15.9% at 0 wt %. The 3 wt % device is also shown to maintain 81% of this efficiency over a period of 480 h aging at 35% RH, compared with only 48% achieved with the 0 wt % device. XPS confirms the homogeneous mixing of the peptide within the MAPI film, with no evidence for an additive-rich overlayer observed, in contrast to our previous studies of perovskite additives.^12,29^ We suggest that this is due to the greater availability of passivating functional sites in the peptide. *In situ* XPS degradation studies show that the threshold for thermally induced decomposition at UHV is raised due to the incorporation of 3 wt % peptide, with some MA^+^ remaining at the surface at 150 °C while the organic cation was completely degraded in the 0 wt % sample. Significantly, and unlike our previous studies of perovskite additives, this thermal stability is partially preserved at 9 mbar water vapor pressure, with some MA^+^ remaining at the surface following the heating regime. We attribute the enhanced water tolerance to the encapsulation of MAPI crystallites by the passivating peptide network inhibiting the migration of ions and reducing the ingress of water molecules. These results suggest that optimization of such peptide-perovskite systems could yield high efficiency, water- and temperature-stable PSCs with tunable optical properties.

## Supporting Information Available

Subtracted FTIR spectrum of MAPI-peptide precursor solution; lattice parameter and cell volume changes determined via Rietveld refinement; details of bandgap calculation using the Brus equation; PL spectra of various peptide concentrations and maximum intensities (log scale); details of time-resolved PL calculations; time-resolved PL spectra fitted with monoexponential decay model; tabulated XPS elemental ratios of 0 and 3 wt % films during heating at UHV; C 1s, O 1s, and N 1s core level XPS spectra from pure FEFKFEFK; XPS survey spectra of pristine 0 and 3 wt % films; overlaid Pb 4f_7/2_, C 1s, and O 1s core level spectra of pristine 0 and 3 wt % films; enlarged N 1s core levels of 0 and 3 wt % films as a function of temperature at UHV and 9 mbar water vapor pressure (PDF)

## Abbreviations

OHP: organometal halide perovskite
MAPI: methylammonium lead iodide perovskite (CH_3_NH_3_PbI_3_)
XPS: X-ray photoelectron spectroscopy
NAP-XPS: near-ambient pressure XPS
MA+: methylammonium cation
PCE: power conversion efficiency
RH: relative humidity
PV: photovoltaic
PSC: perovskite solar cell
ETL: electron transport layer
pHEMA: poly(2-hydroxyethyl methacrylate)
FEFKFEFK: F = phenylalanine, E = glutamic acid, K = lysine
NEXAFS: near-edge X-ray absorption fine structure
VB: valence band
DFT: density functional theory
XRD: X-ray diffraction
PL: photoluminescence
UHV: ultrahigh vacuum
ITO: indium tin oxide
MAI: methylammonium iodide (CH_3_NH_3_I)
HTL: hole transport layer
UV–vis–NIR: ultraviolet–visible–near-infrared
TCSPC: time-correlated, single-photon counting
RT: room temperature
BE: binding energy
RSF: relative sensitivity factor
UPS: ultraviolet photoelectron spectroscopy
UV: ultraviolet
PEY: partial electron yield
TEY: total electron yield
fwhm: full width half-maximum
FTIR: Fourier transform infrared
SEED: secondary electron energy distribution
VBM: VB maximum
DOS: density of states
KE: kinetic energy
*J*_SC_: short circuit current density
*V*_OC_: open circuit voltage
FF: fill factor
