# Supplementary material for: Toward Water-Resistant, Tunable Perovskite Absorbers Using Peptide Hydrogel Additives
Source: ACS Appl Energy Mater. 2024 Sep 13;7(19):8376–90. doi: 10.1021/acsaem.4c01089 (PMC11480935; doi:10.1021/acsaem.4c01089)
Supplement: Supplementary file 1 — ae4c01089_si_001.pdf [file ae4c01089_si_001.pdf]

SUPPORTING INFORMATION

Towards water-resistant, tunable perovskite absorbers using peptide hydrogel additives

Tom Flavell,[ab] Dawei Zhao, [ac] Fahad A. Aljuaid,[ac] Xuzhao Liu,[ac] Alberto Saiani,[de] Alexei B. Preobrajenski,[f] Alexander V. Generalov,[f] Ben F. Spencer,[ag] Alex S. Walton,[ah] Andrew G. Thomas[acg]* and Wendy R. Flavell[ab]

a. Photon Science Institute, University of Manchester, Oxford Road, Manchester, M13 9PL, UK.

b. Department of Physics and Astronomy, University of Manchester, Oxford Road, Manchester, M13 9PL, UK.

c. Department of Materials, University of Manchester, Oxford Road, Manchester, M13 9PL, UK.

d. Manchester Institute of Biotechnology, University of Manchester, Oxford Road, Manchester, M13 9PL, UK.

e. Division of Pharmacy and Optometry, School of Health Sciences, University of Manchester, Oxford Road, Manchester, M13 9PL, UK.

f. MAX IV Laboratory, Lund 221 00, Sweden.

g. Henry Royce Institute, University of Manchester, Oxford Road, Manchester, M13 9PL, UK.

h. Department of Chemistry, University of Manchester, Oxford Road, Manchester, M13 9PL, UK.

* Corresponding author. andrew.g.thomas@manchester.ac.uk

Table of Contents

X-ray diffraction at higher peptide loadings. *S-2*

Fourier transform infrared spectrometry. *S-3*

Conductivity of the perovskite-peptide films. *S-4*

Table of transverse resistance across films. *S-4*

Rietveld refinement from XRD patterns. *S-5*

Bandgaps calculated using the Brus equation. *S-6*

Time-resolved photoluminescence. *S-6*

Photoluminescence (PL) intensity. *S-7*

Calculation of the UV-vis NIR and optical band gap onset. *S-8*

Scanning electron microscopy (SEM) top view. *S-9*

J-V measurements and device performance. *S-9*

Photovoltaic performance data. S-9

Thermal decomposition under UHV conditions measured using X-ray photoelectron spectroscopy (XPS). *S-10*

Comparison of N 1s XPS spectra in UHV conditions and under 9 mbar water vapor pressure. *S-12*

Contributions of the authors. *S-12*

References. *S-13*

X-ray diffraction at higher peptide loadings.

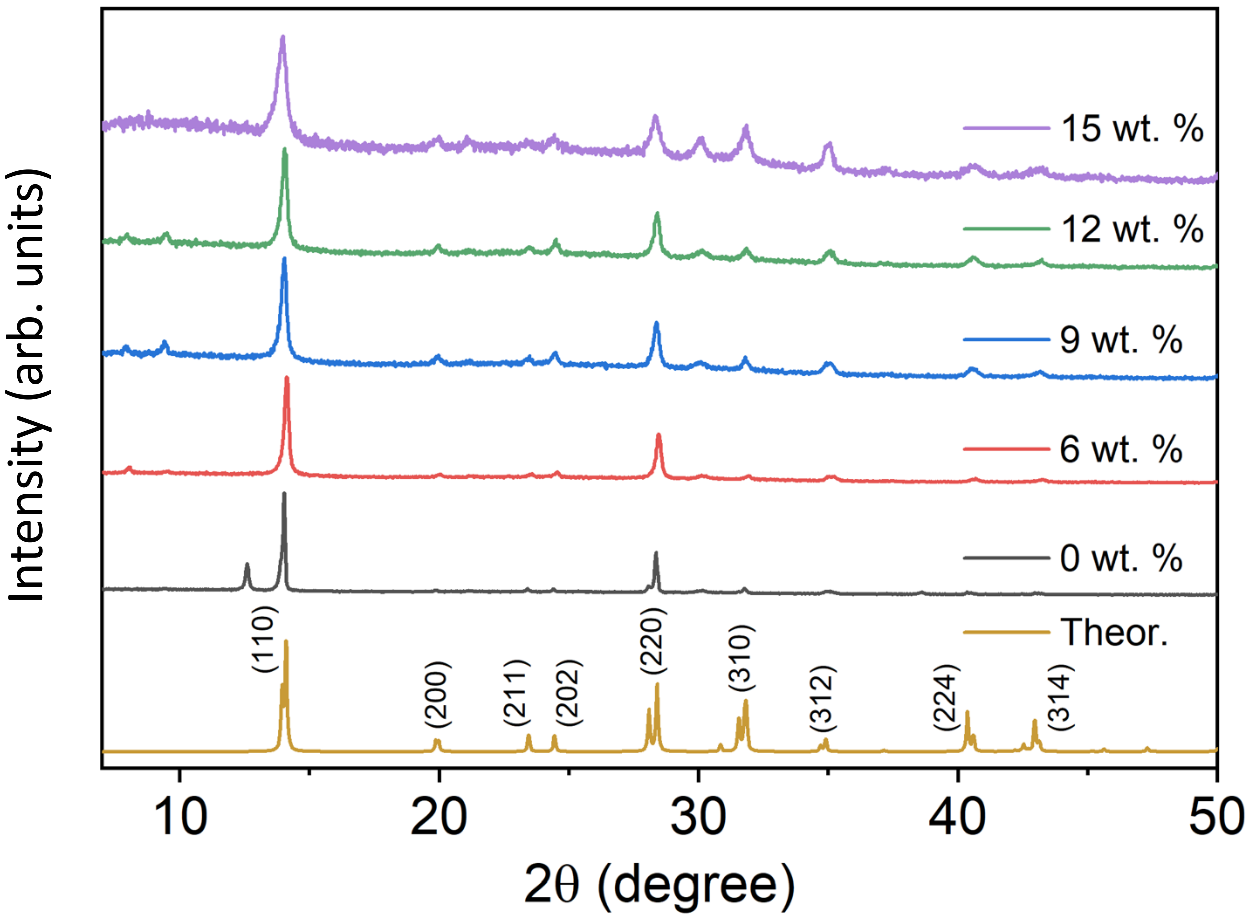

Figure S1. XRD patterns of MAPI containing various 0 – 15 wt % peptide concentration. Clear peak broadening is seen at the higher concentrations, indicating a decrease in crystallite size.

Fourier transform infrared spectrometry.

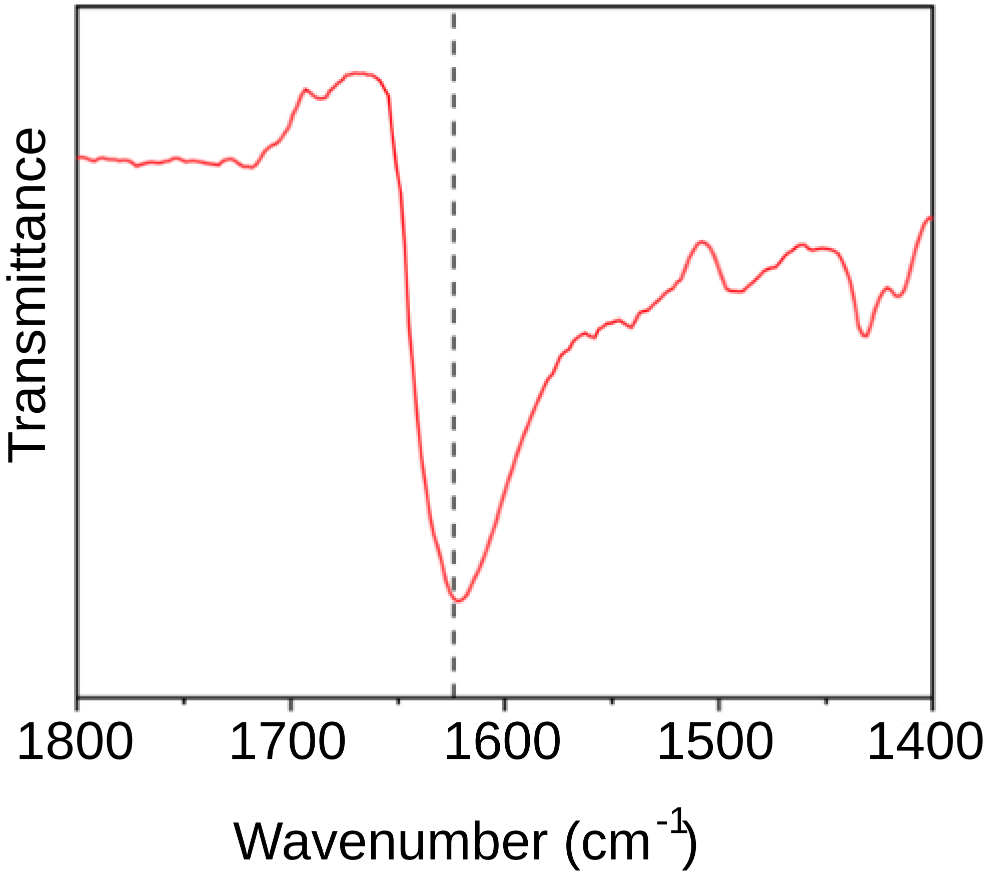

Figure S2. FTIR spectrum of 6 wt. % MAPI-peptide precursor solution, with the spectrum of pure DMF subtracted. The dashed line at 1625 cm^{-1} indicates the position of the absorption band characteristic of the presence of anti-parallel β-sheets.[1] The presence of β-sheets confirms the gelation of the peptide.

Conductivity of the perovskite-peptide films.

Conductivity across the film was measured by the spin coated perovskite onto ITO coated glass and depositing Au electrodes on the top surface. I/V curves were measured as shown below. Very small variations in the conductivity were observed at the peptide loadings measured here.

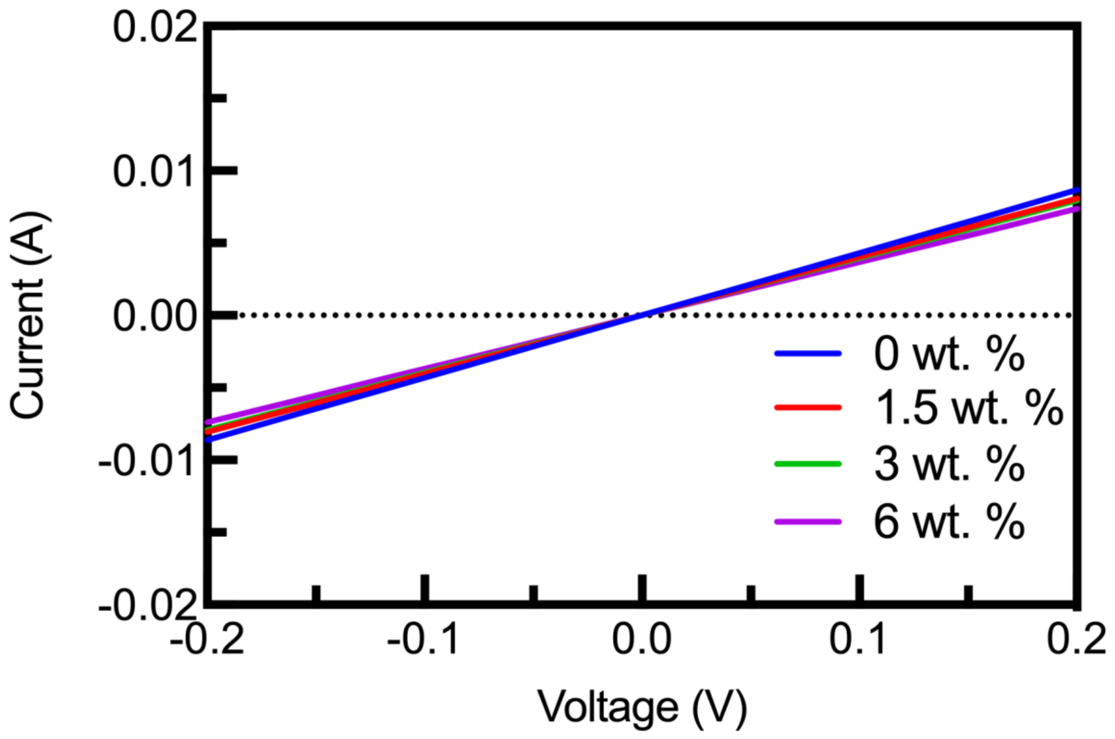

Figure S3. I-V curves of 0 wt.%, 1.5 wt.%, 3 wt.% and 6 wt.% samples based on the configuration of ITO/MAPI/Au

Table of transverse resistance across films.

**Sample**	**Resistance across film (ITO to top contact) (Ω)**
0 wt. %	22.9 ± 0.5
1.5 wt. %	24.7 ± 0.3
3 wt. %	25.2 ± 0.3
6 wt. %	27.0 ± 0.1

Table S1. Transverse resistance i.e. across films (between ITO and top Au contact) calculated from the plots in Figure S7.

Rietveld refinement from XRD patterns.

Rietveld refinement was performed on XRD patterns using DIFFRAC.SUITE TOPAS V.6.0 and a MAPI CIF file (ID: 2107954).[2]

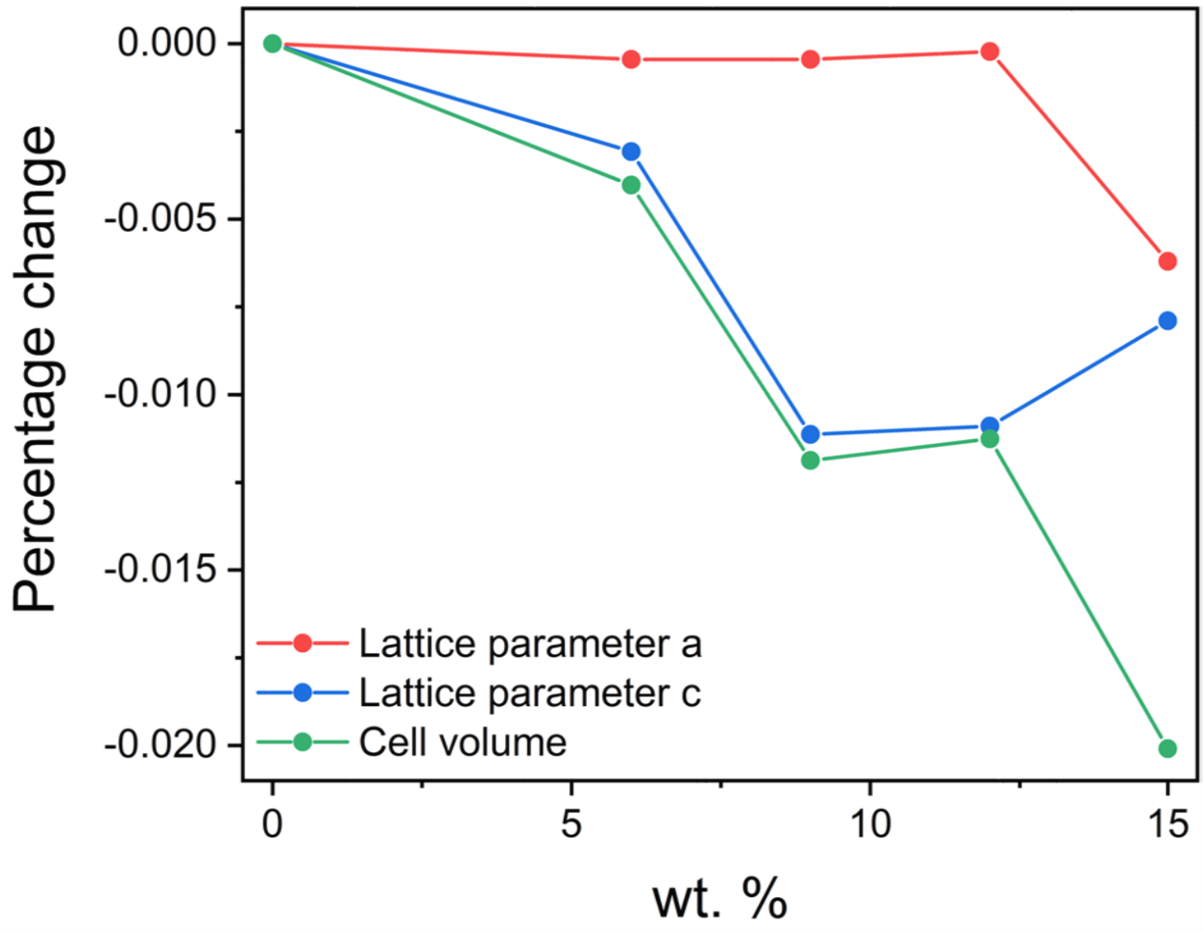

Figure S4. Lattice parameter and cell volume changes as a percentage of those calculated for 0 wt. % for various weight percentages of peptide in the MAPI composite.

Bandgaps calculated using the Brus equation.

To help elucidate whether quantum size effects have any influence on the observed increase in bandgap with increased peptide loading, the expected bandgaps for the crystal radii reported in Table 1 were estimated using the Brus equation (eq. S1).[3, 4]

$$E_{\mathrm{g}}(r) = E_{\mathrm{g}}(\mathrm{bulk}) + \frac{h^2}{8r^2}\left(\frac{1}{m_{\mathrm{e}}{}^*} + \frac{1}{m_{\mathrm{h}}{}^*}\right) \quad (\mathbf{eq.\,S1})$$

Here, the bandgap of a MAPI nanocrystal of radius r, $E_g(r)$, was calculated using the experimentally determined bandgap of bulk MAPI, E_g(bulk) (found to be 1.58 ± 0.01 eV in this study, consistent with previous reports for MAPI[5-7]), and the effective masses of the excited electron and hole, m_e* and m_h* respectively. Here, we use values estimated by Giorgi *et al.*[8], m_e* = 0.23 and m_h* = 0.29.[8] The estimated bandgaps for various loadings of peptide are displayed in the main text in Table 1.

Time-resolved photoluminescence.

Data displayed in Figure 3d was fitted using a double-exponential decay model, defined by eq S2.[9]

$$\mathrm{F(t)} = \mathrm{a_1 e^{-t/\tau_1}} + \mathrm{a_2 e^{-t/\tau_2}} \quad (\mathbf{eq.\,S2})$$

Here, $\mathrm{a_1 e}^{-t/\tau_1}$ and $\mathrm{a_2 e}^{-t/\tau_2}$ correspond to the short and long lifetime components of the fluorescence decay, respectively. Once fitted, the average fluorescence lifetime was calculated using eq S3.[9]

$$\tau = \frac{\mathrm{a_1\tau_1 + a_2\tau_2}}{\mathrm{a_1 + a_2}} \quad (\mathbf{eq.\,S3})$$

Data displayed in Figure S5 was fitted using a mono-exponential decay model, defined by eq S4.

$$\mathrm{F}(t) = \mathrm{a e}^{-t/\tau} \quad (\mathbf{eq.\,S4})$$

Photoluminescence (PL) intensity.

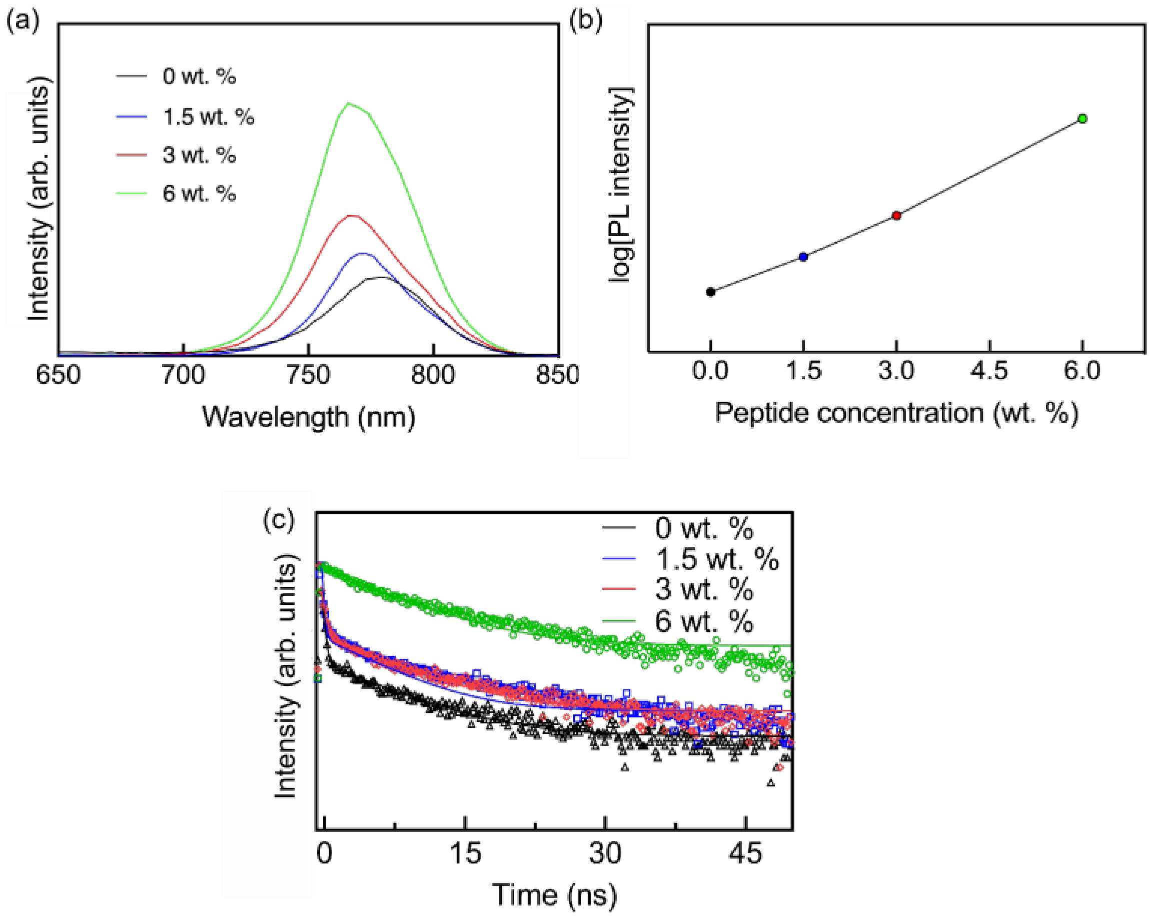

Figure S5. (a) PL spectra of various concentrations of peptide and (b) maximum PL intensity as a function of peptide concentration (log scale), (c) Time-resolved PL spectra for 0, 1.5, 3 and 6 wt% peptide concentrations. The 0, 1.5 and 3 wt. % samples are fitted with a double exponential functions (see above) and the 6 wt.% was fitted with a mono-exponential function

Calculation of the UV-vis NIR and optical band gap onset.

Tauc plots cannot be used to calculate the band gap for these materials since the film thickness may not be uniform, and therefore cannot be quantified at the point on the sample where the UV/visible beam strikes it. In addition, in general for materials with a complex band structure, and in situations where there may also be defect states involved the Tauc method is not suitable to determine the electronic band gap[10]. Here then we determine the optical band gap, simply by taking the onset of absorption, as a tangent to this onset as shown below in Figure S6c.

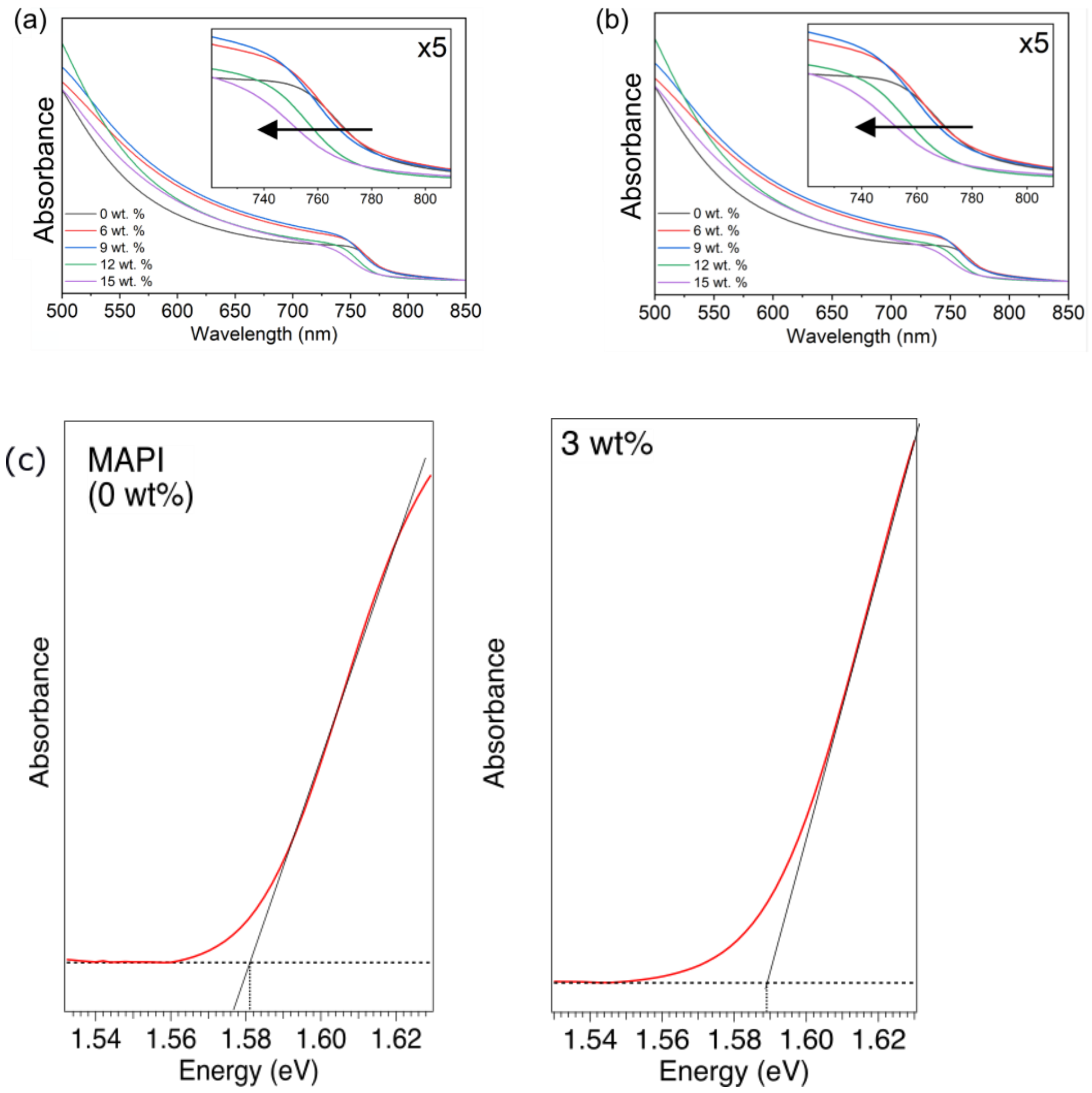

Figure S6. (a) UV-Vis-NIR spectra of MAPI with 0 wt.%, 1.5 wt.%, 3 wt.% and 6 wt.% peptide, (b) with 0 wt %, 6 wt %, 9 wt %, 12 wt % and 15 wt % peptide loading, (c) Example of calculation of optical band gap for pure MAPI and 3 wt% peptide loading.

Scanning electron microscopy (SEM) top view.

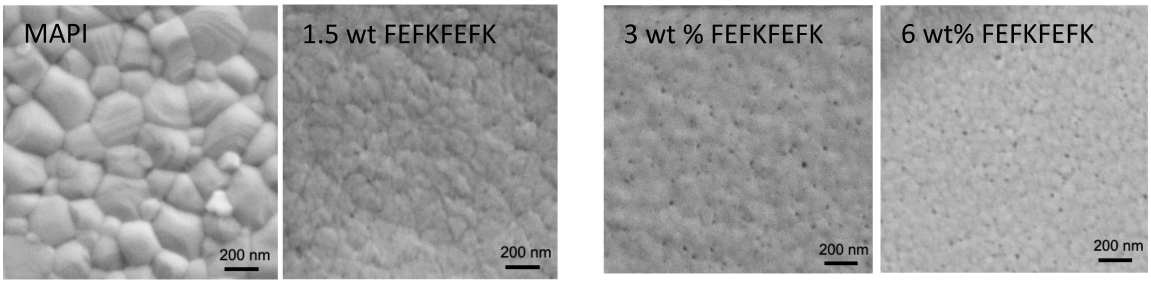

Figure S7. SEM images (top) of MAPI with 0 wt.%, 1.5 wt.%, 3 wt.% and 6 wt.% peptide.

J-V measurements and device performance.

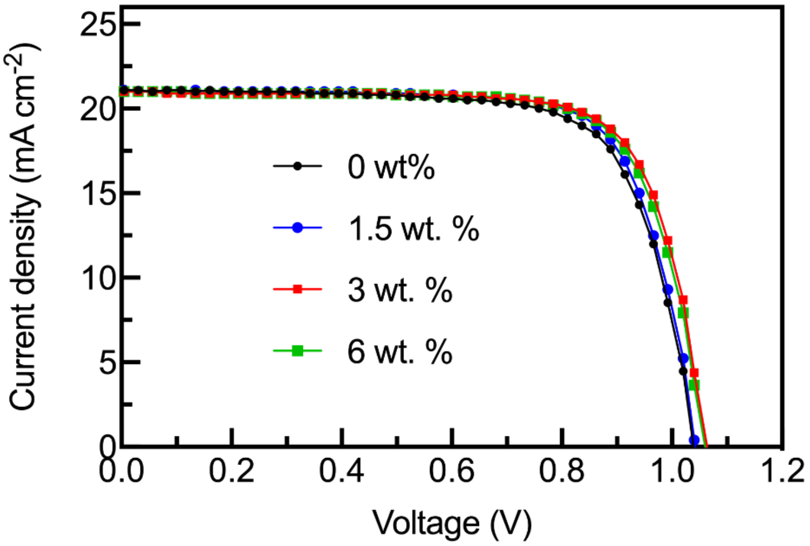

Figure S8. J-V curve of PSCs with active layers containing 0 wt.%, 1.5 wt.%, 3 wt.% and 6 wt.% peptide.

Photovoltaic performance data.

Table S2. Photovoltaic parameters of PSCs with 0 wt. % and 3 wt. % peptide[a]

Sample		J_{SC} (mA cm^{-2})	V_{OC} (V)	FF (%)	PCE (%)
0 wt. %	Champion	21.1	1.04	72.5	15.9
1.5 wt. %	Champion	21.1	1.05	74.1	16.4
3 wt. %	Champion	21.0	1.06	75.1	16.8
6 wt. %	Champion	21.0	1.06	74.6	16.6

Thermal decomposition under UHV conditions measured using X-ray photoelectron spectroscopy (XPS).

Table S3. Elemental ratios in a 0 wt. % MAPI film as a function of temperature. Values were calculated from core level XPS spectra displayed in Figure 7.

0 wt. %			
Temperature	I/Pb^{2+}	N/Pb^{2+}	Pb^0/Pb^{2+}
RT	2.5 ± 0.1	0.6 ± 0.1	0.04 ± 0.01
100 °C	2.4 ± 0.1	0.5 ± 0.1	0.04 ± 0.01
150 °C	1.8 ± 0.1	-	0.03 ± 0.01
180 °C	1.8 ± 0.1	-	0.14 ± 0.01

Table S4. Elemental ratios in a 3 wt. % MAPI-peptide film as a function of temperature. Values were calculated from core level XPS spectra displayed in Figure 7.

3 wt. %				
Temperature	I/Pb^{2+}	N_{MAPI}/Pb^{2+}	Pb^0/Pb^{2+}	N_{pep}/Pb^{2+}
RT	2.4 ± 0.1	0.7 ± 0.1	0.03 ± 0.01	0.4 ± 0.1
100 °C	2.3 ± 0.1	0.7 ± 0.1	0.03 ± 0.01	0.4 ± 0.1
150 °C	2.1 ± 0.1	0.4 ± 0.1	0.05 ± 0.01	0.3 ± 0.1
180 °C	1.8 ± 0.1	-	0.14 ± 0.01	0.2 ± 0.1

C 1s, O 1s and N 1s high-resolution core level XPS spectra from FEFKFEFK deposited on ITO are shown in Figure S4. The sample was prepared by mixing 80 mg mL^{-1} FEFKFEFK in DMF solvent, which was deposited onto an ITO substrate and heated at 100 °C until the solvent evaporated, to closely mimic the fabrication process of the MAPI samples. XPS was conducted at RT under UHV conditions. The C 1s spectrum consists of three distinct peaks at 284.8 ± 0.1, 286.3 ± 0.1 and 288.0 ± 0.1 eV BE, assigned to C-C / C=C, C-N, and O=C-N / O=C-O respectively.[11-14] The O 1s spectrum contains two peaks corresponding to the carboxyl (C=O) and hydroxyl (OH) oxygen at 531.8 ± 0.1 eV and 533.7 ± 0.1 eV BE respectively.[13] The N 1s spectrum displays a strong peak at 400.0 ± 0.1 eV BE, assigned to the amide bond in the peptide.[10-12] The smaller peak at 401.5 ± 0.1 eV BE is attributed to positively charged lysine side chains, NH_3^+.[12, 13]

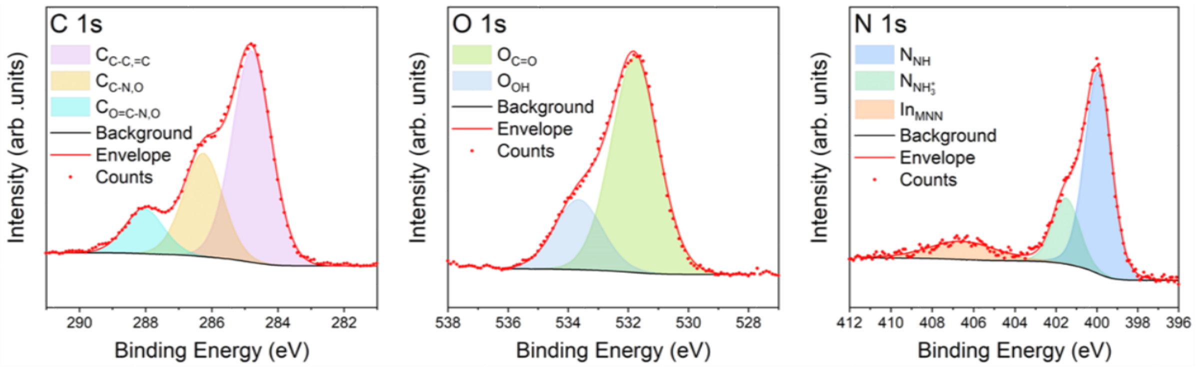

Figure S9. N 1s and C 1s high-resolution core level XPS spectra of peptide deposited on ITO with fitted peaks labelled. The N 1s spectrum contains an Auger peak (In_{MNN}) originating from the ITO substrate. The C 1s peak labelled $C_{C-C,=C}$ also contains a contribution from adventitious carbon. The spectra were calibrated to the C-C / C=C peak at 284.8 eV.

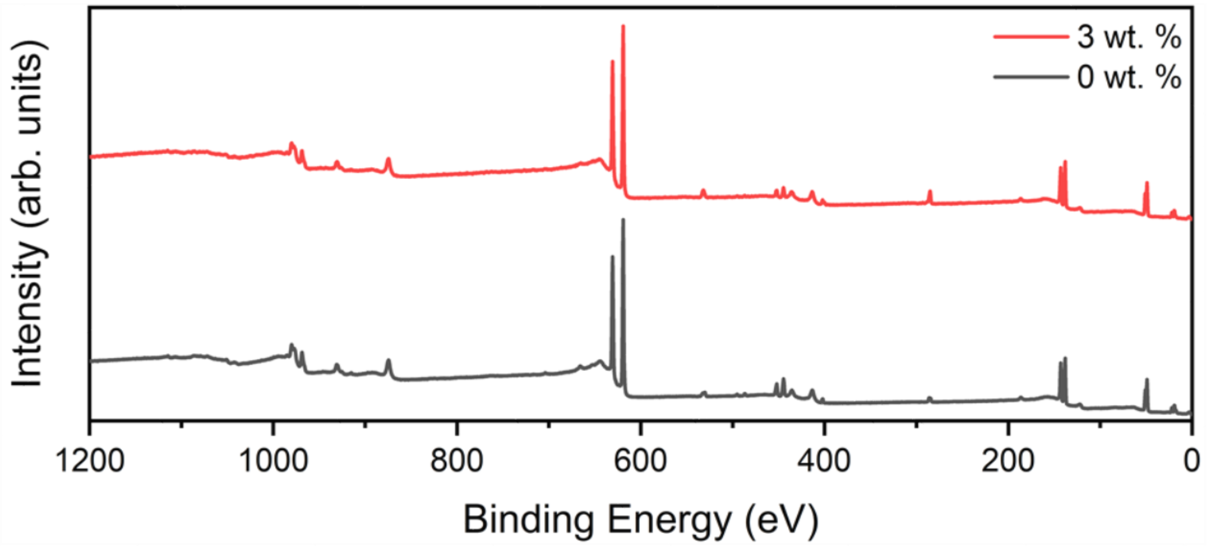

Figure S10. XPS survey spectra of pristine 0 wt. % and 3 wt. % MAPI-peptide films, showing no noticeable difference in inelastic background shape.

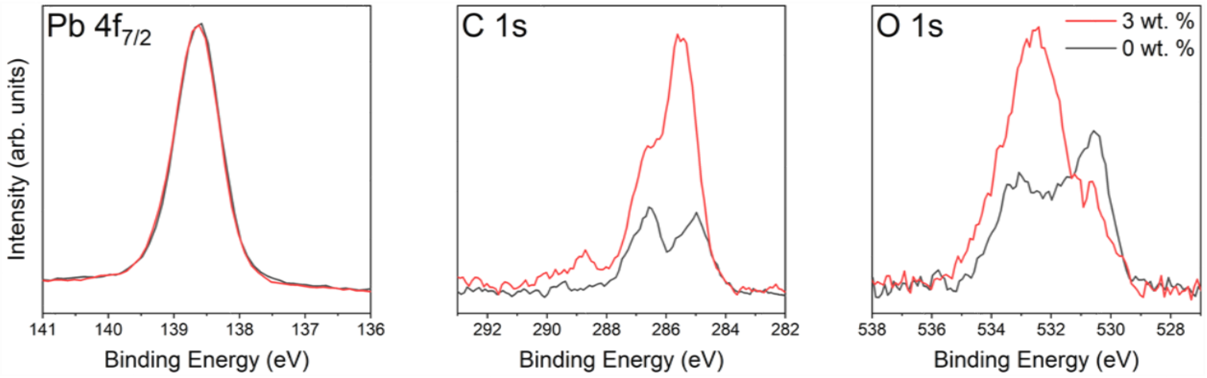

Figure S11. Overlaid Pb $4f_{7/2}$, C 1s and O 1s (left to right) high-resolution core level XPS spectra from 0 wt. % (black) and 3 wt. % (red) MAPI-peptide films, measured at room temperature (RT). Spectra are normalized to the intensity of the Pb 4f peak.

Comparison of N 1s XPS spectra in UHV conditions and under 9 mbar water vapor pressure.

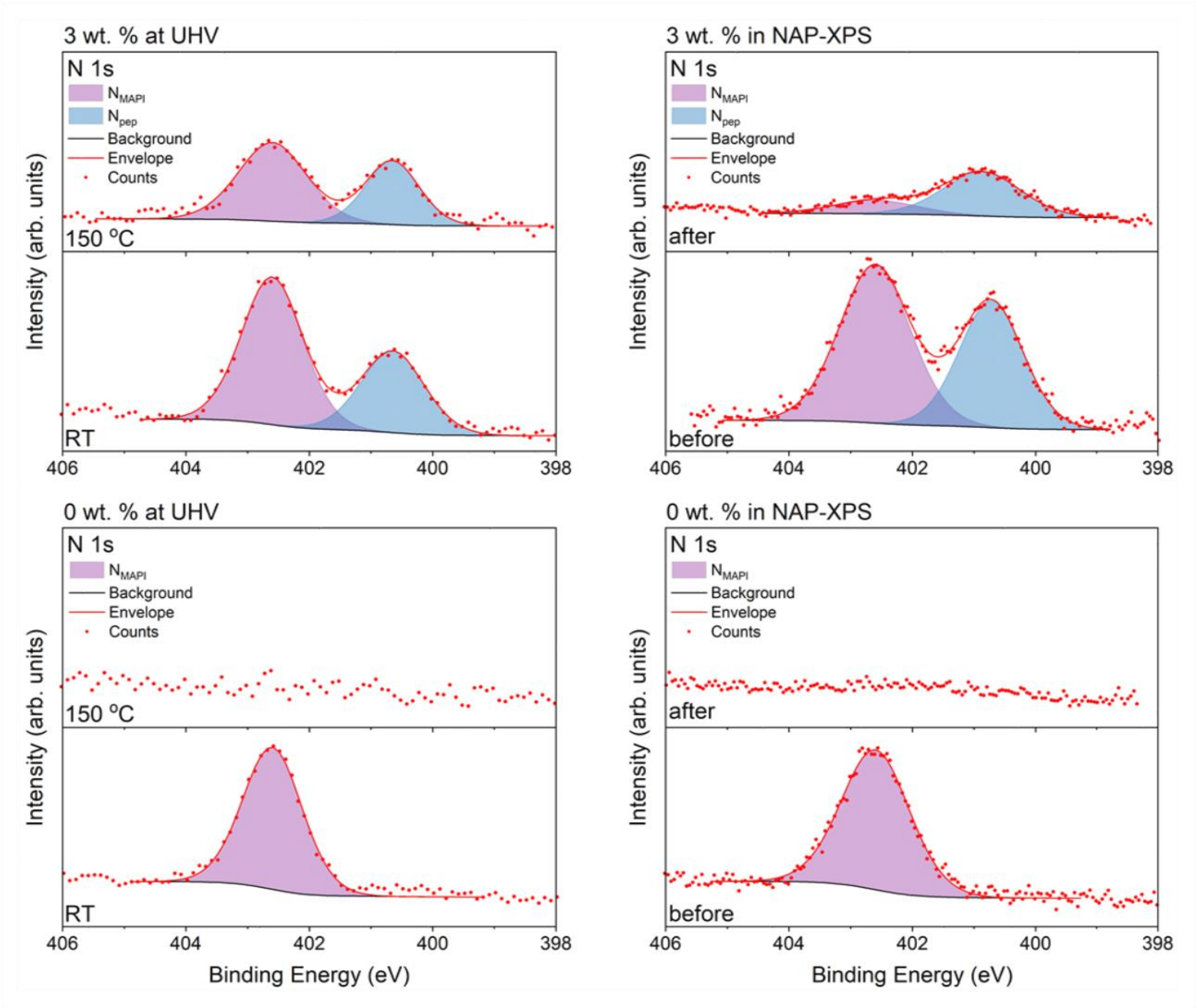

Figure S12. Enlarged N 1s core level region of 0 wt. % and 3 wt. % MAPI-peptide films using XPS at UHV (left) and NAP-XPS at 9 mbar water vapor pressure (right) with fitted peaks labelled. For the NAP-XPS spectra, 'before' and 'after' correspond to UHV measurements at RT before and after the heating regime to 150 °C at 9 mbar water vapor pressure. No clear features are present in the 0 wt. % spectrum following the heating regime at either UHV or 9 mbar, therefore no fitting is included. Dashed lines represent the position of peaks prior to heating and any shift is noted.

Contributions of the authors.

Manuscript preparation, DZ and TF; Materials synthesis and characterisation DZ, XL and FA; XPS measurements and data analysis, AT, TF, WRF; Technical assistance with XPS, NEXAFS, BS, AW, AP, AG; Manuscript editing and approval, All authors.

References.

1. A. Saiani, A. Mohammed, H. Frielinghaus, R. Collins, N. Hodson, C. Kielty, M. Sherratt and A. Miller, *Soft Matter*, 2009, **5**, 193-202.
2. A. Arakcheeva, D. Chernyshov, M. Spina, L. Forró and E. Horváth, *Acta Crystallographica Section B: Structural Science, Crystal Engineering and Materials*, 2016, **72**, 716-722.
3. L. E. Brus, *The Journal of chemical physics*, 1984, **80**, 4403-4409.
4. Y. Kayanuma, *Physical Review B*, 1988, **38**, 9797.
5. E. M. Miller, Y. Zhao, C. C. Mercado, S. K. Saha, J. M. Luther, K. Zhu, V. Stevanović, C. L. Perkins and J. van de Lagemaat, *Physical chemistry chemical physics*, 2014, **16**, 22122-22130.
6. B. Philippe, B.-W. Park, R. Lindblad, J. Oscarsson, S. Ahmadi, E. M. Johansson and H. Rensmo, *Chemistry of Materials*, 2015, **27**, 1720-1731.
7. P. Schulz, L. L. Whittaker ‐ Brooks, B. A. MacLeod, D. C. Olson, Y. L. Loo and A. Kahn, *Advanced Materials Interfaces*, 2015, **2**, 1400532.
8. G. Giorgi, J.-I. Fujisawa, H. Segawa and K. Yamashita, *The journal of physical chemistry letters*, 2013, **4**, 4213-4216.
9. W. Becker, *Advanced time-correlated single photon counting applications*, Springer, 2015.
10. Klein, Julian, Laura Kampermann, Benjamin Mockenhaupt, Malte Behrens, Jennifer Strunk, and Gerd Bacher. *Advanced Functional Materials* 33, (2023): 2304523.
11. R. Konradi, B. Pidhatika, A. Mühlebach and M. Textor, *Langmuir*, 2008, **24**, 613-616.
12. G. Polzonetti, C. Battocchio, G. Iucci, M. Dettin, R. Gambaretto, C. Di Bello and V. Carravetta, *Materials Science and Engineering: C*, 2006, **26**, 929-934.
13. J. S. Stevens, A. C. De Luca, M. Pelendritis, G. Terenghi, S. Downes and S. L. Schroeder, *Surface and Interface Analysis*, 2013, **45**, 1238-1246.
14. Y. Zubavichus, M. Zharnikov, A. Shaporenko, O. Fuchs, L. Weinhardt, C. Heske, E. Umbach, J. D. Denlinger and M. Grunze, *The Journal of Physical Chemistry A*, 2004, **108**, 4557-4565.
